# Detection of Emerald Ash Borer Infestations in Living Green Ash by Noninvasive Electronic-Nose Analysis of Wood Volatiles

**DOI:** 10.3390/bios9040123

**Published:** 2019-10-13

**Authors:** A. Dan Wilson, Lisa B. Forse, Benjamin A. Babst, Mohammad M. Bataineh

**Affiliations:** 1 Pathology Department, Southern Hardwoods Laboratory, Southern Research Station, USDA Forest Service, Stoneville, MS 38776, USA; lbforse@gmail.com; 2 Arkansas Forest Resources Center, and College of Forestry, Agriculture and Natural Resources, University of Arkansas at Monticello, Monticello, AR 71656, USA; Babst@uamont.edu (B.A.B.); Bataineh@uamont.edu (M.M.B.)

**Keywords:** *Agrilus planipennis*, early tree-infestation detection, electronic nose (e-nose), plant-health biomarkers, insect-infestation biomarkers, sapwood, smellprint signatures, VOC-metabolites

## Abstract

The emerald ash borer (EAB) has been the most destructive and costly nonnative insect to threaten the health of ash (*Fraxinus*) species in North America for at least the past 25 years. The development of methods for detecting visually-hidden EAB galleries at early stages of infestation would provide a useful tool to more effectively facilitate the planning and implementation of targeted EAB pest-suppression and management activities. We tested the efficacy of using a dual-technology electronic-nose (e-nose)/gas chromatograph device as a means for detection of EAB infestations in green ash trees in different EAB-decline classes by analysis of VOC emissions in sapwood. We found significant differences in VOC profiles for trees from the four decline classes. The VOC composition, quantities, and types of volatile metabolites present in headspace volatiles varied considerably across sample types, and resulted in distinct e-nose smellprint patterns that were characteristic of each unique chemical composition. In addition, specific VOC metabolites were identified as potential healthy and EAB-infestation biomarkers, indicative of the health states of individual trees. Few significant differences in major bark phenolic compounds were found between ash decline classes using LC-MS. The e-nose was effective in discriminating between uninfested and EAB-infested trees based on sapwood VOC emissions.

## 1. Introduction

The early detection of invasive insect pests in living trees, prior to the occurrence of external visible signs and symptoms of damage, is essential for effective forest and urban pest-management treatments. Stem-boring insects are particularly difficult to detect and control with the absence of visual indicators, especially before significant internal damage results in tree mortality and economic losses. Developing an effective strategy for early pest detection, such as for many xylophilous insect-borer species that cause damage to trees, is often important in the production of hardwood trees grown for quality lumber or other high-valued forest wood products. Wood-boring insects frequently cause internal damage due to the production of larval galleries that contribute to tree mortality, lumber degrades and cull losses in numerous hardwood species [1]. Nonnative invasive insects are usually managed through containment measures to stop or slow spread which depend on an accurate determination of where pest insects are located [2]. Early and accurate detection also informs decision-makers whether to attempt eradication, which is highly dependent on determining the geographical boundaries of the pest population. Furthermore, early detection is essential for establishing quarantine boundaries for effective containment and integrated management of all nonnative invasive insects.

Emerald ash borer (EAB), *Agrilus planipennis*, is a nonnative invasive insect pest that originated in eastern Asian hardwood forests and most likely was accidently introduced into the U.S. in the early 1990s in southeast Michigan (near Detroit), although not officially first detected and recognized as a nonnative pest in that state until 2002 [3]. The larvae of this green-metallic buprestid beetle are phloem feeders and all North American ash species are suitable larval hosts, including black ash (*F. nigra*), white ash (*F. americana*), and green ash (*F. pennsylvanica*) [4]. In Europe, EAB was first discovered in green ash species planted in Moscow, Russia in 2003, subsequently spreading 460 km over the following decade aided primarily by human activity and causing extensive mortality to indigenous European ash (*F. excelsior*) [5]. Subsequent spread of the insect in Europe has resulted in EAB largely eradicating *Fraxinus* species from many member states [6].

The economic and ecological impacts of EAB are quite high and geographically extensive. The spread of this aggressive borer has been extraordinarily rapid, primarily due to human transport of infested ash wood, bark chips, and nursery stock of ash species; and has killed billions of ash trees in forest, riparian, and urban areas over the past 25 years, making it among the most destructive and costly forest insects to invade North America [7,8]. As of 2018, EAB had spread to 35 US states and five Canadian provinces [9]. The U.S. Forest Inventory Analysis (FIA) database indicates that forest lands in the lower 48 U.S. states contain approximately 8.7 billion ash trees and saplings, accounting for about 2.5% of above-ground forest carbon mass [10]. Ash trees, particularly including cultivars of green ash and white ash, are popular landscape trees that comprise more than 20% of the urban forest canopy in some areas [11,12]. Projected costs of treating or replacing only 45% of landscape ash trees having EAB infestations in urban areas were estimated at $10.7 billion USD from 2009 to 2019 [7,13]. Subsequent estimates through 2020 (including landscape trees) could exceed $12.5 billion [14]. Further costs of EAB to municipalities and private property owners could exceed $1 billion USD annually [3].

The EAB is not known to be a serious pest of ash trees in its native Asian range, but only occasionally attacks ash trees that are stressed, damaged, or dying [15,16]. This suggests that Asian ash species have effective defenses against EAB when trees are healthy and not stressed. However, EAB infests and attacks healthy trees in all stages of growth in susceptible North America ash tree species, which suffer high mortality within several years due to larval infestation and destruction of the phloem, cambium, and occasionally the outer-most sapwood during gallery formation [16,17,18,19]. EAB is now widely present in most of the eastern United States, but has not significantly affected lumber values of ash species (due to downgrades of lumber quality) if dead trees are salvaged quickly, because borer galleries usually do not penetrate deeply into the sapwood [20].

Ineffective early EAB-detection methodologies have hampered attempts at early management interventions. For example, the range of initial quarantine zones were likely too narrow to capture the full area already colonized by EAB [21]. Currently, detection of EAB is based on adult trapping with chemical/pheromone attractants and observations of canopy decline and other symptoms, as well as signs such as the diagnostic presence of D-shaped adult exit holes [22]. These methods are costly, time consuming, and are not consistently effective for detection before EAB populations have already become firmly established and caused significant damage to trees. Pheromone traps can be useful, but trap attractants apparently work only at close range and must be properly located near EAB populations to be effective [23]. EAB attractants also are somewhat nonspecific, except for a lactone (3*Z*-dodecen-12-olide) [24,25]. Previous studies indicate that North American ash species have limited biochemical response in leaf and bark tissues to EAB infestation [19,26,27]. Consequently, laboratory-based chemical analysis of tree-tissue samples is not likely a practical means for EAB detection, in addition to the relatively high expense and the time required to obtain lab-test results.

Electronic-nose (e-nose) devices have been used to detect a wide range of insect and disease pests that cause damage to agricultural crops and forest trees [28,29]. Recent applications of e-nose instruments for detection of insect damage and pests include detection of tea loopers (*Ectropis obliqua* and *E. grisescens*) on tea (*Camellia sinensis*) [30], citrus fruit infestations by the Oriental fruit fly (*Bactrocera dorsalis*) [31], red palm weevil (*Rhynchophorus ferrugineous*) [32], rice infestations by striped rice stem borer (*Chilo suppressalis*) and brown rice plant hopper (*Nilaparvata lugens*) [33,34], and stink bug infestations of cotton bolls [35,36]. An e-nose also has been used to detect acarinid damage, such as for spider mite (*Tetranychus urticae*) infestations of greenhouse cucumbers [37].

Early detection of EAB larval feeding in the sapwood of *Fraxinus* species could provide a much-needed tool to aid EAB pest management. The purpose of this study was to investigate the potential efficacy of utilizing an e-nose technology for early detection of EAB-infestations at early stages, prior to the appearance of diagnostic symptoms in the tree crown and adult emergence holes in the lower bole, by using green ash as an initial test species in southern Arkansas. Our specific objectives were to: 1) develop effective, noninvasive sampling methods, potentially associated with periodically-timed plantation or forest-stand pest-damage surveys, for collecting VOC emissions within air samples derived from sampled wood cores for e-nose analyses, 2) establish e-nose based aroma signature (smellprint) patterns of wood-core volatiles from nonsymptomatic, uninfested trees and those in various stages of decline due to EAB infestations (ash decline classes) to develop an e-nose reference library (for assessing the health states of individual trees), 3) determine the potential capabilities and efficacy of a specific dual-technology E-nose/GC instrument for discriminating between uninfested and EAB-infested trees based on VOC smellprint signatures, and 4) assess the effects of EAB larval infestations on production of induced host-defense VOCs in sapwood by E-nose/GC analysis and non-volatile compounds in the bark using LC-MS.

## 2. Materials and Methods

The geographical distribution of EAB infestations of ash trees initially was confirmed for six southwestern Arkansas counties in 2014. The range was extended to 23 confirmed counties in the state as of September 2019. The current research study site is located in the northeastern corner of Clark County, which is among the first six counties where EAB was confirmed in Arkansas. The study site is 1.6 km east of Arkadelphia, AR, where EAB was confirmed at this site in August 2016. Research plots of green ash test trees were established in an ash-tree plantation containing 25–40-year-old trees within a frequently-flooded, forested wetland area with bottomland hardwood stands. The test sites were in a 0–2% sloped stream-terraced area with deep, poorly-drained soils composed of fluvial sediments of silt loam, belonging to the Foley soil series, located near the floodplain of the Ouachita River in the South Central Plains Region. All test sites had a similar management and disturbance history.

### 2.1. Field Sampling of Wood Cores

Wood radial increment cores were collected from 43 green ash trees for chemical analyses. Test tree samples were collected 8 June 2017, and tree crown and status ratings were scored on 25 May 2017. All samples for chemical analyses were taken from live, standing green ash. We collected a total of 86 sapwood cores and bark samples from young trees, with narrowly variable diameter at breast height (dbh) and tree heights at four crown health rating classes (1 through 4; Table 1), based on scorings of crown declines due to EAB with corresponding associated levels of EAB sapwood-infestation status as previously described [38,39]. All samples were weighted to 0.01 g using a Sartorius LC 6200S MC-1 digital scale (Sartorius Corp., Göttingen, Germany).

Bole tree-core samples (0.5 × 6.5 cm) were taken from the sapwood of live green ash trees from each crown health class with a two-thread increment borer (Haglöf Inc., Långsele, Sweden) at or near breast height above the ground. Similarly, bark samples were collected using modified wood chisels and mallet at or near breast height above the ground.

### 2.2. Sample Preparations for GC/E-nose Chemical Analyses

Sapwood core samples were held frozen at –20 °C until analyzed. Frozen samples were thawed to 22 °C, soaked in 18.2 mΩ pure water (Millipore Model Milli-Q UV-Plus, Molsheim, France) for 30 s, blotted dry, and placed individually into a 100 glass-sampling bottles with lids sealed with a 4.3 cm PTFE-faced Silicone septum (Pyrex, Corning, Corning, NY, USA).

All samples were heated in a Model 750F oven (Fisher Scientific, Pittsburg, PA, USA) at 35 °C for 60 min to build headspace volatiles. Samples were allowed to cool to 22 °C just prior to GC/E-nose analysis. Sample VOC emissions in sample-bottle headspace were taken by withdrawing two 15 mL aliquots of separate replicate injections for GC/E-nose analyses.

### 2.3. GC/E-nose Configuration Parameters and Data Acquisition

The Heracles II GC/Electronic-nose system (Alpha MOS, Toulouse, France), utilized for all tree core VOC-headspace analyses, was composed of a dual-column (DB-5 and DB-1701) fast-gas chromatograph (GC) with hydrogen carrier gas, two flame-ionization detectors (FID), and multi-sensor e-nose consisting of hundreds of metal-oxide semiconductor (MOS) type sensors. The 32 best sensors contributing to discrimination of sample types were determined for all VOC-sample classes, based on pre-statistical evaluations of data. These specific, most-discriminating sensors were employed in developing smellprint signatures of each aroma class (sample type). Operational gases (hydrogen carrier and oxygen for FID detectors) were generated using an Alliance Desktop Hydrogen Generator system PAR.H2.180.V3 (MicroProgel Srl, Torreglia, Italy).

Headspace volatiles derived from tree samples were manually withdrawn using a 20 mL glass syringe (Cadence Science Inc., Cranston, RI, USA) and 15 mL of headspace gas from each sample was injected into the system, first passing into a Peltier-cooled adsorption cold trapped at 30 °C for 50 s before entering into 10 m, 0.18 mm-diam. dual GC columns (DB-5 and DB-1701) via split-injection at 10 mL/min. This was followed by isothermal heating at 240 °C for 30 s at 57 kPa of pressure. Analyses were conducted at an initial oven temperature of 50 °C, ramping at 1 °C/s up to 80 °C, then accelerating the heating rate to 3 °C/s up to 250 °C for 21 s. Analyzer injection volume was set at 5000 μL at a speed of 125 μL/s, injection temperature of 200 °C at 10 kPa pressure, injection time 45 s, and venting at 30 mL/min. FID detector temperatures, for two separate dual columns, were set at 260 °C. Retention times (RTs) and peak areas of VOCs from each sample type were recorded for each peak for both GC columns up to the maximum run times of 110 s. Dual-column GC-data outputs provided opportunities to help resolve VOC-peaks that had close or overlapping RTs.

GC-output data were collected during analyses along with the full range of VOC-peak information for all sample types and replications, which were determined by calculation. Integrated peak areas (under the curve) were used rather than peak heights due to highly variable GC-peak shapes. Actual mg quantities of VOCs present in individual peaks were not determined, since quantitative standard curves using analytical standards for specific VOCs were not established. Data acquisition rates for both GC data recording and e-nose data from the sensor array were collected every 0.01 s intervals (100 data points per second) set at a constant data-recording rate for the entire duration of each analysis run. Both dual GC-columns and e-nose sensor arrays were purged with ultrapure zero-air or blank samples between each sample analysis run to prevent carryover of VOC sample-analytes between runs. Multiple purge runs with ultrapure zero-air also were run before and after sample-run sequences. 

The e-nose analyzer component of the dual-technology Heracles II system utilizes a very large number of proprietary MOS sensors in the sensor array. For data analyses, the thirty-two sensors providing the largest output responses facilitating sample discriminations were utilized for all data analyses. Smellprint patterns, defined for each aroma class, consisted of assembled sensor-response intensity outputs for all sensors in the sensor array that responded to all VOC analytes present in complex VOC-mixtures for each sample class. Smellprints were determined as unique chemical aroma signatures of VOC-metabolite mixtures contained within headspace volatiles of individual sample classes.

### 2.4. Tree Bark Solvent Extraction Preparation for LC-MS Analysis

Ultra-high-performance liquid chromatography-mass spectrometry (LC-MS) bark samples were frozen in liquid N_2_ and stored at −80 °C until processed. Lyophilized bark samples (10 mg) were extracted twice in 500 µL ice cold 100% methanol (MeOH) for 10 min in a Bransonic ultrasonic bath (Danbury, CT, USA) maintained at 4 °C. The extracts were centrifuged at 18,000 g for 2 min at 4 °C to remove particulates, and supernatants were combined and filtered through a 0.45 µm filter prior to injecting 1 µL of methanolic extract for each LC-MS run.

### 2.5. LC-MS Analysis Methods

Analysis of methanolic bark-extract samples by LC-MS was done using an Ultimate 3000 UHPLC with UV diode array detector (Thermo Scientific) coupled with an LTQ XL linear ion trap MS (Thermo Scientific, San Jose, CA, USA) with heated electrospray ionization. Samples were kept at 10 °C in an autosampler prior to injection during LC-MS analysis, and the injection needle was washed with 100 µL 10% MeOH between each injection. Samples were separated by a 10 cm × 2.1 mm inner diameter Accucore Vanquish C18+ column with 1.5 µm particle size, kept at 35 °C using a column oven, using mobile phase A: 97% water 3% acetonitrile with 0.1% formic acid, and mobile phase B: 97% acetonitrile 3% water with 0.1% formic acid. The elution gradient was 3% B for the first minute, linear gradient to 12% B during 1–3 min, linear gradient to 17% during 3–7 min, linear gradient to 30% B from 7 to 15 min, linear gradient to 97% B from 15 to 16 min, isocratic at 97% B until 21 min, at which point the mobile phase was returned to 3% B and allowed 3 min to re-equilibrate before injecting the next sample.

Compounds were detected by recording full spectrum absorbance between wavelengths 200–399 nm at 5 Hz, and by mass spectrometry (MS). Molecules were ionized for MS by electrospray ionization (ESI) with the following settings: heater temperature: 225 °C, capillary temperature: 315 °C, sheath gas: 75, auxiliary gas: 5, sweep gas: 0, spray voltage: 3750 V, capillary voltage: −27 V, tube lens: −132.8 V. The LC stream was diverted to waste for the first 1 min to avoid contaminating the MS with compounds that did not interact with column and separate from one another. The MS was set to negative ionization mode. MS2 scans, which fragment one of the primary ions and provide the mass spectrum of the resulting fragment ions, were used to assist with compound identification. Each scan cycle included five scan events, including four data-dependent MS2 scans, (1) a full MS scan from m/z 50 to 1500 AMU, (2) MS2 scan of the most intense ion from the full scan (event #1), (3) MS2 scan of the second most intense ion from the full scan, (4) MS2 scan of the third most intense ion from the full scan, and (5) MS2 scan of the fourth most intense ion from the full scan. For MS2 scans, the minimum threshold signal was set to 500 counts, and fragmentation was achieved by CID activation (collision-induced dissociation) with normalized collision energy set to 35, activation Q set to 0.25, activation time 30 ms, default charge state of 1, and isolation width of m/z 2.0 AMU. Rates for all MS and MS2 scans were set to “normal.” UV and MS2 spectra were used primarily for compound identification.

### 2.6. Statistical Analysis of GC/E-nose and HPLC Data

Statistical analyses of e-nose, smellprint signatures, principal component analysis (PCA), and discriminant factor analysis (DFA) data were carried out using Alphasoft v14.20 and AroChembase software using methods described previously [40]. Peak area data from GC chromatograms were analyzed by one-way analysis of variance (ANOVA), Brown-Forsythe Equal Variance Test, and Fisher least significant difference (LSD) tests using SigmaPlot v14.0 software. Three-dimensional PCA was performed on e-nose data to compare the relatedness between uninfested, nonsymptomatic (healthy), and EAB-infested green ash cores based on aroma signature patterns derived from e-nose sensor array output responses to wood core VOC-metabolite mixtures in headspace. Highly unrepresentative outlier data of each sample type were removed as necessary to improve statistical models for effective sample discriminations. Distances between centers of data clusters (PCA mapping distance), derived from sensor array outputs of each wood core headspace from each EAB decline-class sample type (aroma classes), were determined on a PCA plot or aroma map by pairwise comparisons of aroma classes in all possible combinations, along with aroma Pattern Discrimination Index (PDI), expressed as a percentage approximating the statistical level of discrimination (P-values) between corresponding sample types compared, based on calculated differences in aroma signature patterns (smellprints). Comparisons between smellprint signatures of VOCs from sapwood cores from the four tree crown heath classes (nonsymptomatic, light, moderate, and heavy infestations by EAB-larvae) were done using 3-dimensional DFA to determine the effects of varying EAB-infestation levels, correlated with associated crown health ratings, on differences in mixtures of VOC-metabolites in sapwood emissions. Variations in mean sensor response intensities (MSRIs) of individual sensors to replicate samples of each aroma class, displayed within smellprint signatures, were calculated and displayed as means ± 1 standard deviation (SD) from the mean indicated by shaded areas on bar graphs.

Data derived from LC-MS chemical analyses were preprocessed using TraceFinder™ 4.1 software (Thermo Scientific, San Jose, CA, USA). A qualitative, non-targeted pre-screening was first performed to determine m/z and retention times of ions that were present in bark-extract samples using a ICIS peak-detection algorithm with a minimum MS signal threshold of 10,000, mass tolerance of 500 mmu, smoothing set to 5, minimum peak signal to noise ratio of 50, and a minimum/maximum peak width of 0.08 and 1.0 min, respectively. When the aligned results of the screening (for all of the samples combined) were curated to remove redundant ions, 192 unique ions were identified, although some compounds were subsequently determined to be represented by several ions such as [M-H]−, formide adducts ([M+CHOOH-H]-), and electrospray-induced dimers. The 192 curated ions and their retention times were used to conduct a targeted quantification in the TraceFinder 4.1 software, using ICIS peak detection algorithm with smoothing set to 5, a 30 s retention time window, and other parameters adjusted as needed for each ion to achieve acceptable peak integration that was uniform across all samples. Every peak was checked visually, and the integration adjusted manually, if necessary. For each compound, peak areas for each symptomatic crown-decline class (2–4) were compared with peak areas for healthy trees (1) using a Student’s t-test.

## 3. Results

The results of chromatographic and e-nose data presented here are limited to chemical analyses of low molecular-weight VOC emissions from sapwood cores and nonvolatile heavier molecular-weight metabolites from bark extracts.

### 3.1. Electronic-nose Analysis of Sapwood Core Volatiles

**Figure 1:**
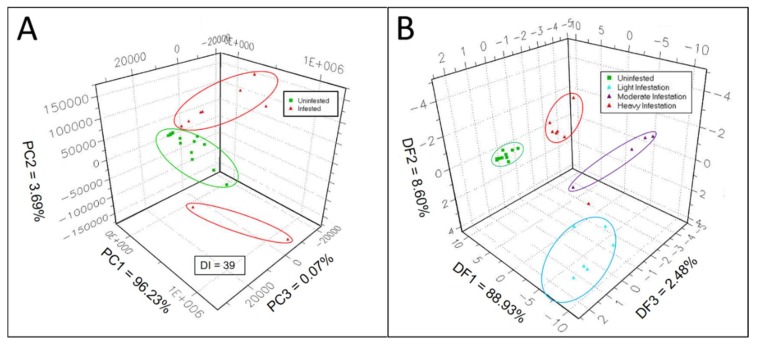
Discrimination between VOC-metabolite profiles of green ash sapwood cores with different levels of EAB infestation. Three-dimensional factor analysis of e-nose aroma classes by: (**A**) Principal component analysis (PCA) between uninfested (healthy) sapwood and EAB-infested sapwood; (**B**) discriminant factor analysis (DFA) between sapwood cores from different tree crown health classes corresponding to various levels of EAB sapwood infestation. The Discrimination Index (DI) value for 3-d PCA was statistically valid at P < 0.05 level of significance.

Sensor responses of the e-nose sensor array to complex gaseous mixtures of volatile metabolites, released into sampling-chamber headspace, were analyzed statistically using 3-d PCA to measure and quantify differences between e-nose aroma signatures produced in response to VOCs released from healthy and EAB-infested sapwood. A 3-d PCA aroma map data plot of uninfested (healthy, represented by green squares) vs. the three combined EAB-decline classes, subgroups with various levels of EAB-infestations (represented by red triangles), showed the distribution of data clusters with relative chemical relatedness between aroma classes of VOC-metabolite emissions from green ash sapwood cores (Figure 1A). This PCA aroma map of all sample types tested provided a visual representation for comparing PCA mapping distances between data clusters of each sample type, as well as a Discrimination Index (DI), indicating the relative overall strength or level of discrimination between all sample types included in the PCA test. The displayed DI value, validated by Alphasoft V14.20 software (using green highlighting), indicates a passing PCA test at P ≤ 0.01 level of significance when a statistically-successful 3-d PCA discrimination has been achieved between sample types (mapped data clusters). The percentages of total variance, accounting for the variability explained by each orthogonal principal component in the PCA, are as follows: PC 1 = 96.23%; PC 2 = 3.69%; and PC 3 = 0.07%. Thus, most of the variability in the PCA was accounted for by PC 1 (x-axis), whereas PC 2 (y-axis) and PC 3 (z-axis) accounted for only a minor proportion of the total variance.

The plotted e-nose aroma data cluster for VOCs from uninfested green ash sapwood cores was well separated from combined data clusters for VOCs from sapwood cores taken from trees with the three levels of EAB-infestation (light, moderate, and heavy). The overall discrimination index was DI = 39 (statistically valid) for this 3-d PCA plot analysis. There were two data points, discovered as outliers derived from two heavily-infested sapwood core samples, that putatively were taken in very close proximity to an EAB larval gallery from heavily EAB-infested trees. VOC emissions from cores represented by these two outliers were found to be chemically different from sapwood core VOC-emissions from all other heavy-infestation core samples, putatively taken at a greater distance from a larval gallery. VOC emissions from outlier cores contained additional induced VOCs probably derived directly from larvae and frass sources within EAB galleries. Data from GC analyses of core VOC emissions, derived from both constitutive and infestation-induced volatiles, provided additional evidence identifying two chemically-distinct VOC-emission types (groups) from heavily infested sapwood cores (see GC-peak area data in Section 3.1 and Section 3.2).

The 3-d DFA data plot, comparing data clusters of VOC e-nose smellprint signatures from volatile metabolites derived from cores of the four EAB-decline sample types (in various stages of EAB larval-infestation or subgroups), provided a means for visually indicating differences in chemical relatedness between VOC-metabolite mixtures derived from sapwood headspace volatiles for each e-nose aroma class or sample type (Figure 1B). Data clusters representing VOC-metabolites from sapwood sample types with different levels of EAB-larval infestation were well separated in 3-dimensional space with no overlaps between data clusters. Again, there was a single outlier data point for one of the sapwood cores from the heavily infested sample type. The data cluster of the uninfested (healthy, represented by green squares) sample type was the smallest and tightest (least variable) cluster among the four sample types. The most widely dispersed and largest data cluster occurred with the light EAB-infestation sample type (cyan-colored triangles), whereas data clusters of the moderate (magenta-colored triangles) and heavily infested (red triangles) sapwood were intermediate in size and spatial distribution. The breakdown of total variance percentages, accounting for data variability explained by each orthogonal principal component in the DFA, are as follows: DF 1 = 88.93%; DF 2 = 8.60%; and DF 3 = 2.48%. Consequently, the majority of the variability in the DFA was accounted for by DF 1 (x-axis), whereas DF 2 (y-axis) and DF 3 (z-axis) accounted for only minor proportions of the total variance.

DFA distances between centers of data clusters of the DFA aroma plot map, determined from pairwise comparisons of aroma-class data clusters, yielded indications of absolute chemical differences in VOC-metabolite mixtures of aroma classes represented by distinct, color-coded data clusters. This information provided a metric with an exact quantitative and statistical indicator, via pattern discrimination index (PDI) percentage differences, of chemical relatedness between aroma classes as presented in Table 2. These results indicated that the biggest differences in chemical relatedness between headspace volatiles (highest DFA distances and PDI%) occurred in comparisons of the uninfested sample class with the three levels of EAB-infestation sample classes.

These significant differences of > 93% PDI, recorded for all three pairwise comparisons, suggested high levels of e-nose discrimination between VOC-metabolites of healthy and infested sapwood sample types. The lowest DFA distances were found between the light and moderate EAB-infestation sapwood samples, although still with high levels of difference and discrimination at a PDI percentage of 90.1%. Intermediate levels of chemical differences (PDI ≈ 92%) were found between VOC-metabolites of light- and severe-infestation sample types and between moderate- and severe-infestation aroma-sample classes.

**Figure 2:**
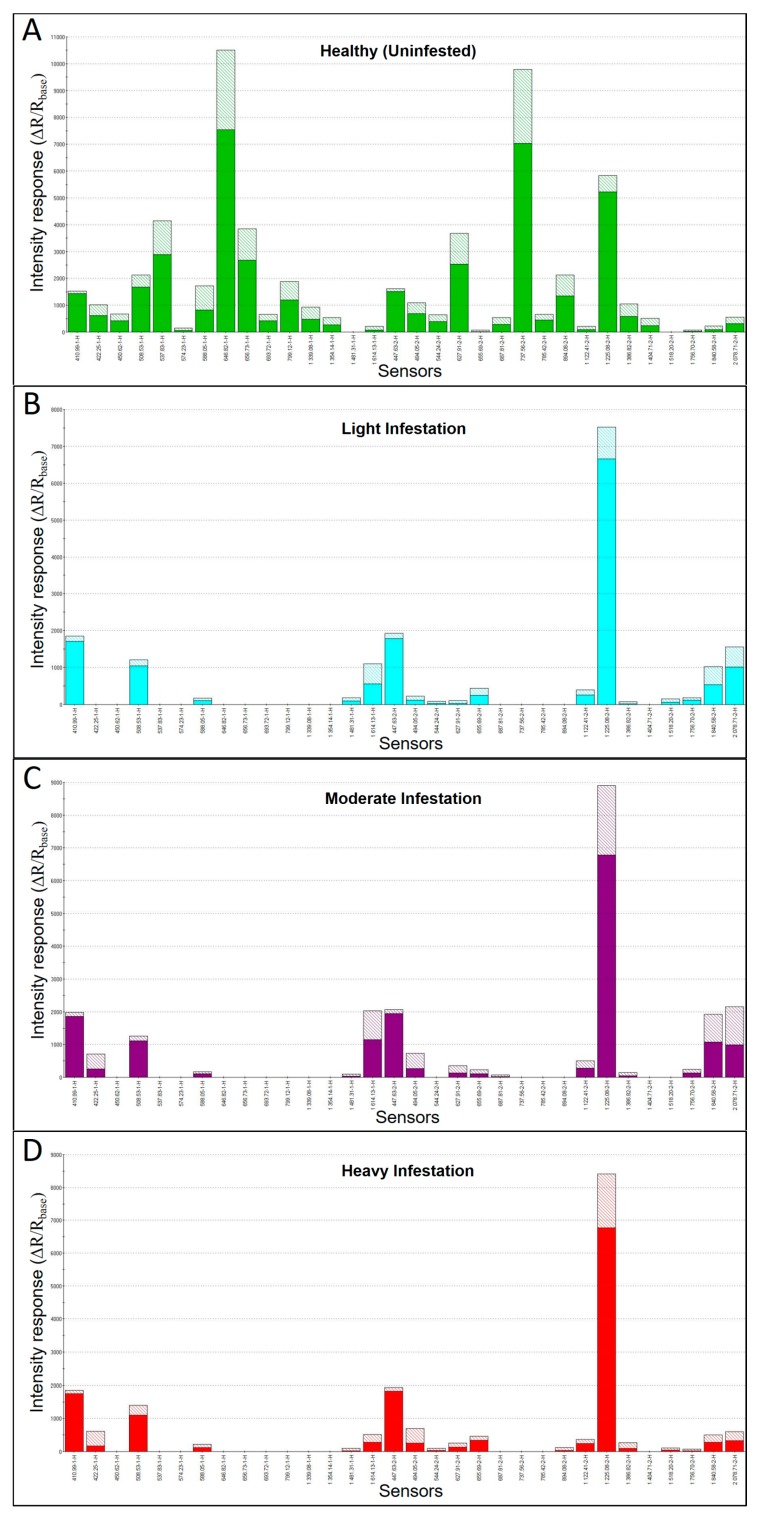
Electronic-nose smellprint signatures of VOC-metabolites from green ash sapwood cores with different levels of EAB infestation. (**A**) Smellprint signature of healthy (uninfested) sapwood (decline rating 1); (**B**) Smellprint signature of sapwood with light EAB-infestation level (decline rating 2); (**C**) Smellprint signature of sapwood with moderate EAB-infestation level (decline rating 3); (**D**) Smellprint signature of sapwood with heavy EAB-infestation level (decline rating 4). Shaded areas indicate one standard deviation from the mean.

Additional MOS e-nose analysis of headspace VOC metabolites derived from green ash sapwood cores, based on sensor-intensity responses for the top 32-sensors within the sensor array providing the greatest discrimination between sample types, produced sensor response patterns (smellprints) that varied significantly with relative levels of EAB-infestation of trees from different crown health-class categories associated with EAB-decline (Figure 2A–D). Sensor responses of the same 32 sensors producing the best discrimination of samples types yielded quite different smellprint signatures indicating chemical differences between VOC emissions from sapwood cores of trees with different EAB-decline ratings and corresponding levels of sapwood infestations. VOC-emissions from uninfested (healthy) sapwood cores (decline rating 1) induced a broad and relatively strong sensor-intensity response of all MOS sensors across the selected array, with the exception of no responses for sensor S-14 (1481.31-1-H) and sensor S-29 (1518.20-2-H). Sensor S-8 (646.82-1-H) had the strongest intensity response (10.5k) of all sensors to healthy sapwood VOC-metabolites. Sensor S-22 (737.56-2-H) had the second highest strong-intensity response (9.8k) of all sensors responding to healthy sapwood VOC-metabolites.

Analysis of smellprint signatures of VOC-metabolites from EAB-infested sapwood cores produced considerably different results from uninfested cores. Almost half (13 to15, or 41–47%) of all sensors in the selected array had no responses to VOC-metabolites from EAB-infested cores, regardless of decline rating or level of sapwood infestation. The strongest sensor intensity response (range 7.5–8.9k) to VOC emissions from cores taken from separate trees with all three levels of EAB-infestation (decline ratings 2–4) occurred with MOS sensor S-26 (1225.08-2-H). The remaining ≤ 29 sensors in the array produced relatively low sensor-intensity responses (≤ 2k) to EAB-infested sapwood cores.

Variability in individual sensor responses, among the three levels of EAB-infested cores, were notably observed for differences in sensors that had no responses and in differences in relative intensity of responses of specific sensors to changes in VOC-metabolite emissions for each decline rating with associated different levels of EAB infestations.

**Figure 3:**
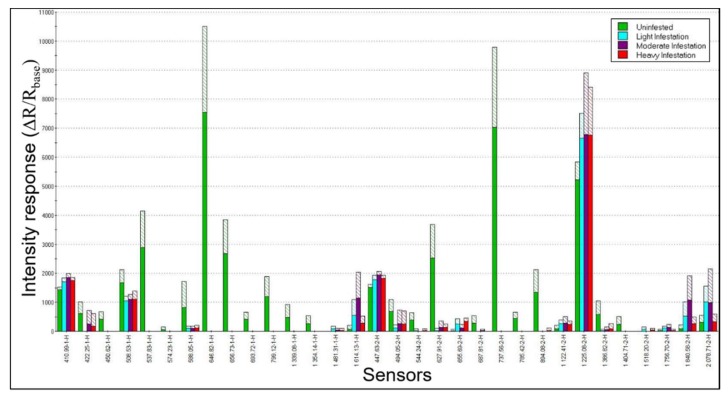
Combined e-nose smellprint signatures of VOC-metabolites from green ash sapwood cores with different levels of EAB infestation. Smellprint signatures patterns derived from the 32-sensor array responses to: (1) Healthy (uninfested) sapwood (decline rating 1, green bars); (2) light EAB-infestation level (decline rating 2, cyan bars); (3) moderate EAB-infestation level (decline rating 3, magenta bars); (4) heavy EAB-infestation level (decline rating 4, red bars). Shaded areas indicate one standard deviation from the mean.

A comparison of combined smellprint signatures resulting from simultaneous analysis of VOC metabolites from sapwood cores of trees from all four crown class ratings (different aroma classes) yielded a VOC smellprint profile showing more clearly the differences in sensor-response intensities of individual sensors in the array for all EAB-decline sample types (Figure 3). The most obvious difference in smellprint signatures between samples types is the abundance of sensor responses to VOC-metabolites from healthy tree cores (green bars) that are absent in EAB-infested trees. Twelve separate sensors responded only to volatiles from healthy tree sapwood cores, whereas only two sensors, including sensor S-14 (1481.31-1-H) and sensor S-29 (518.20-2-H), responded only to VOCs from EAB-infested sapwood cores and had no responses to VOCs from healthy sapwood cores.

Comparisons of differences in sensor-response intensities to VOC-emissions from healthy and EAB-infested cores (from trees in various stages of EAB-decline) provided data relating to changes or shifts in VOC production over time, as healthy trees become infested and infestation levels in sapwood increase proportionally to more advanced stages of decline. This comparison is possible because sensor-response intensities are semi-quantitatively correlated with concentrations of VOCs present in sapwood-core headspace. In the majority of cases (60%), sensor response intensities to VOC-emissions from EAB-infested sapwood were greater than the same sensor-responses to VOCs from healthy sapwood when sensor responses (15 of 32 total sensors) occurred for all four sample types analyzed. The rare instances where VOC-emissions from healthy cores were greater than those from all infested cores occurred only for six sensors, including sensor S2 (422.25-1-H), S4 (508.53-1-H), S7 (588.05-1-H), S17 (494.05-2-H), S19 (627.91-2-H), and S27 (1386.82-2-H).

### 3.2. Gas Chromatographic Analysis of Sapwood Core Volatiles

The analysis of GC chemical data from the dual-column fast-gas GC portion of the e-nose provided information relating to the relative quantities and numbers of VOCs that comprised the complex headspace volatile emissions from green ash sapwood core sample types of each decline rating, simultaneously analyzed with the MOS e-nose sensor array. The major peaks and associated retention times (RTs) of VOCs present in volatile emissions from cores of each crown health class (decline rating) are presented with peak areas in Table 3. The relative quantities of VOC emissions for each sample type are grouped by sequential peak number and metabolomic clusters. 

Metabolomic clusters were defined by whether constitutive VOCs produced by both healthy and EAB infested sapwood had metabolomic changes in relative quantities of VOCs produced. The groupings of sensor clusters were based on whether the peak areas were uniform, decreasing, or increasing in quantities of VOC-emissions from cores of EAB-infected trees relative to uninfested (healthy) control cores. Four constitutive VOCs (peaks 4, 5, 6, 13) were identified with uniform peak areas for both healthy and infested cores. Two VOCs (peaks 2 and 3) decreased in peak area for cores from infested trees (relative to healthy trees) with the exception of near-gallery severely-infested cores. Two additional VOCs (peaks 1, 9) generally increased (statistically) in VOC peak areas for infested tree cores (derived presumably from host-induced or insect-derived sources) compared to cores from healthy trees. Four VOCs (peaks 7, 8, 11, 12) also had uniform peaks areas for infested cores, compared to healthy, except for putative near-gallery cores. The two cores defined as putative near-gallery cores (a subtype among the severe decline samples), showed exceptionally high VOC emissions (> 6 to 15-fold increase) compared to those of healthy trees and were considered outliers.

Other VOC emissions, produced differentially by sapwood cores of the four health class (decline-rating) categories, were identified and clustered sequentially by peak number into three categories based on emission source, including the first group (healthy host VOCs) that were only released from cores of healthy trees, a second group (nonspecific VOCs) not apparently constitutively produced (not always produced) nor induced by infestation, and a third group of VOCs which were produced only in cores of EAB-infested trees (decline ratings 2–4). The major peaks and RTs of VOCs present in volatile emissions from cores of each crown health class are presented with peak areas, based on sample type and emission-source clusters, in Table 4. Eight VOC emissions (peaks 1, 2, 4–6, 8, 12, 13) were exclusively released only from healthy (previously uninfested) host trees, but stopped being produced in sapwood cores once EAB-infestation was initiated, thus not constitutively-produced tree VOC emissions. Two VOC emissions (peaks 3, 7) from a nonspecific (unidentified) source were occasionally produced in cores from both healthy and all EAB-infested sample types, but were not consistently produced by either group. Five VOC emissions (peaks 9–11, 14, 15) were identified only from infested cores and were usually absent, with only occasional trace-quantities found in healthy cores. This third group of VOCs was presumed to be composed of either EAB infestation-induced host VOCs within sapwood or of insect origin, composed of insect-derived VOCs from EAB larvae or frass within galleries. The occurrence of higher peak areas in specific cores from among those taken from severely declining trees, putatively collected near larval galleries, provided additional evidence that VOCs from this group were induced by EAB-infestations that resulted in higher infestation-induced host emissions or from direct insect derivation nearer larval galleries. The production of these apparent infestation-induced or insect-derived VOC emissions occurred in cores from all categories of EAB-infested trees cores, with the exception of VOCs represented by peaks 9 and 11, which exhibited no emission of these VOCs from some sample types among infested cores.

#### Chemical Identities of VOC-Emissions Derived from Sapwood Cores

The tentative identities of host-derived constitutive VOCs identified from analysis of GC peaks, previously identified from Table 3, were determined using RT-values, Kovats Retention Indices (KRIs), Relevance Indices (RI), eleven aliphatic alkane internal Kovats reference standards, and a >83,000-organic compound reference library for comparisons based on nearest matches to KRI-values of known VOCs, which are presented in Table 5. The list of tentative identities of constitutive VOCs were from a wide range of chemical classes.

The five constitutive VOCs (peaks 4, 5, 6, 13), identified with uniform peak areas from both healthy and infested cores, were identified as aliphatic diketones and esters, carboxylic acids, sulfhydril ketone, lactone, and bicyclic sesquiterpenes. Two VOCs (peaks 2 and 3) that showed decreased emission in cores from infested trees relative to healthy trees tentatively were identified as aliphatic aldehydes, ketones, and alkanes.

Two VOCs (peaks 1 and 9) that increased in infested cores relative to healthy cores were identified as a methyl ester and bicyclic monoterpene, respectively. Four additional VOCs (peaks 7, 8, 11, 12) which had uniform peak areas for healthy and infested cores, but much higher peak areas for putative near-EAB gallery cores, were identified as volatile, bicyclic monoterpenes, aliphatic alkanes, esters, cyclic monoterpenes, homocyclic ethyl esters, and possibly pyran monoterpenes.

The most notable major constitutive VOC (peak 7), largest in abundance based on peak area, was among those uniformly produced in all infested cores, but increased significantly in putative near-EAB-gallery sapwood cores, and was tentatively identified as most probably (1S)-(-)-α-pinene (a bicyclic monoterpene). The second largest constitutive VOC (peak 2) by area which decreased in infested cores compared to healthy was identified as propanal (an aliphatic aldehyde). The third largest VOC (peak 11) was identified as limonene (a cyclic monoterpene), while the fourth largest VOC (peak 8) was identified as either 3-ethyloctane or 3-methylnonane (both aliphatic alkanes) or amyl-propanoate (an aliphatic ester). The identity of peak 12, produced in lesser quantities but still at significantly high emissions, curiously was identified as either ethyl cyclohexane-carboxylate (a homocyclic ethyl ester) or cis-rose oxide (a pyran monoterpene). Two additional constitutive VOCs, rarely produced in sapwood of healthy and EAB-infested trees, included a peak at RT = 53.47 s, tentatively identified as aliphatic aldehydes E,E-2,4-hexadienal or methional, and a peak at RT = 60.79 s, tentatively 1-methylpropyl benzene or 2-ethyl-3-methyl-pyrazine (a pyrazine derivative).

The tentative chemical identities of source-specific VOCs, produced only by sapwood of healthy hosts or only from EAB-infested sapwood due to either infestation-induced host response or direct insect-derived emissions as previously identified in peak clusters specified in Table 4, are presented in Table 6. The eight VOC emissions (peaks 1, 2, 4–6, 8, 12, 13) exclusively detected from healthy (previously uninfested) host trees, but not produced in EAB-infested cores, were tentatively identified as aliphatic alcohols or thiols, nitriles, aldehydes, esters, ketones, and alkanes. The two VOC emissions (peaks 3, 7), which were not specific to certain crown classes but were only occasionally produced in cores from both healthy and all EAB-infested sample types, were identified as aliphatic and heterocyclic thiols. The five VOC emissions (peaks 9-11, 14, 15) identified only from EAB-infested cores were identified as aromatic esters, monoterpenes, aliphatic esters, bicyclic sesquiterpenes, and monosequiterpene alcohols. 

The most significant VOC emissions exclusively produced from EAB-infested cores were tentatively identified for peak 9 as 6-decenal (an aliphatic aldehyde) or methyl salicylate (an aromatic esters) and peak 14 as butyl salicylate (aromatic ester) or β-caryophyllene (a bicyclic sesquiterpene), peak 10 as nerol (a monoterpene), peak 11 as hexyl 2-butenoate (an aliphatic ester). Finally, peak 15 was identified as α-bisabolol oxide A (a monocyclic sesquiterpene alcohol) or caryophyllene acetate (a bicyclic sesquiterpene ester). One additional VOC emission specific to EAB-infested sapwood was discovered at RT = 67.89 s, identified as either n-nonanal (an aliphatic aldehyde), p-menthatriene (a monocyclic diene), or (±)-cis-rose oxide (a pyran monoterpene), which was not produced in healthy sapwood. All of the VOC emissions limited to infested cores were less volatile and higher molecular weight compounds than most healthy-specific VOCs, having RTs >72 s and KRIs >1200 for a 10 m DB-5 capillary column.

### 3.3. HPLC Analysis of Methanolic Bark Extracts

LC-MS analysis detected 192 nonredundant ions in the mass spectrum of green ash bark methanolic extracts, including some of the major compounds previously identified in green ash bark, such as verbascoside, ligustroside, and oleuropein (Table S1). However, some previously identified compounds were not abundant enough to be detected in our study, such as hydroxytyrosol hexoside and quercetin diglycoside.

There were only 16 highly abundant (signal intensity >10^6^ ) nonvolatile, polar phenolic compounds found by LC-MS analysis of bark extracts, which are presented in Table 7. Nine of the most abundant compounds (represented by peaks 9, 12, 28, 58, 66, 72, 88, 103, and 149) were significantly affected by EAB infestation, and eight of these decreased in abundance as a result of EAB infestation. Only one of the highly abundant compounds (peak 16) increased modestly (yet significantly) in abundance in the bark of only moderately-declining EAB-infested trees.

We were unable to identify ten of the major bark phenolics (represented by peaks 9, 12, 16, 20, 24, 28, 58, 66, and 139), including most of the lower molecular weight compounds (RTs = 1.3–9.2 min) due to the lack of matches with any known compounds in the MS-database. Two phenolics, (peaks 72 and 88) with RTs = 9.9–10.8 min, decreased in abundance in the early decline stage (class 2) and matched the m/z of the parent and MS2 fragment ions of verbascoside, a defense biochemical in ash phloem. Two other peaks (107 and 127) had the fragment ion 461 in common with verbascoside, and could be a verbascoside isomer and derivative, respectively. One phenolic (peak 149) matched the mass spectral characteristics of ligustroside. Peak 103 matched the mass spectrum of oleuropein, and another phenolic (peak 154) was consistent with oleuropein hexoside.

The majority (118, or 61.5%) of the 192 total compounds extracted from green ash bark were not significantly affected by EAB infestation, regardless of decline class (Table S1). Of the 55 compounds (28.6% of the total) affected by EAB infestation, most decreased in abundance (Table S2). Only 19 compounds increased in abundance in EAB-infested trees, and 14 of those only increased in one decline class.

## 4. Discussion

Electronic-nose analysis of VOC-emission profiles from tree cores of uninfested and EAB-infested trees (in various stages of decline) using PCA indicated significant differences in the composition of headspace volatiles from sapwood before and after EAB attack. Two distinct PCA data clusters (representing VOC-emissions from infested and uninfested) were observed, along with a smaller subgroup of outlier samples from heavily-infested trees, which were putatively derived from cores taken near or immediately adjacent to larval galleries. VOC emissions from the outlier heavily-infested sample types contained additional induced VOC components, likely derived directly from larval and/or frass sources. The majority of other cores taken from heavily-infested trees, presumably taken further away from larval galleries, lacked these additional induced VOC emissions. Subsequent GC-chemical analysis of VOCs provided strong evidence from peak areas, indicating that VOCs derived from putative near-gallery cores occurred at significantly higher concentrations than were observed in all other infested cores from different decline classes.

Analysis of VOC-emission profiles from sapwood cores of all four decline classes, previously defined as an FIA ash-crown condition rating scale modified by Smith [38,39], by DFA showed that the chemical composition of VOC emissions was significantly different for the four sample types. VOC-emissions from healthy cores formed a tight data cluster, whereas the data clusters for the three decline classes (with different levels of EAB-infestation) were separate, but more diffuse. Pairwise comparisons of data clusters between all four decline classes showed high levels of differences in chemical relatedness between VOC emissions as confirmed by PDI values > 90% differences between all combinations of decline sample types.

Smellprint signatures, derived from e-nose sensor-array response intensity patterns, of VOC emissions from sapwood of healthy trees consisted of strongly-positive responses for most sensors across the array. By contrast, the smellprint signatures of VOC emissions from sapwood cores of lightly infested, moderately-infested, and heavily-infested trees were superficially very similar and exhibited a sparse sensor response pattern and only weakly positive or no responses for most sensors in the array. These results suggested a much wider diversity of VOC composition and higher concentrations (abundance or quantities) of VOC emissions from healthy vs. EAB-infested sapwood.

The emissions of a wider range of VOC types and greater quantities in healthy sapwood compared with emissions from sapwood of infested trees is most likely explained by host-induced shifts in metabolic pathways away from some normal anabolic activities to production of chemically and structurally related compounds for wound healing and perhaps ineffective host-defenses. Previous research has suggested that trees initiate inducible defenses in four stages in response to insect and disease attacks by downregulation of gene expression and energy resources devoted to primary metabolic activities, and upregulation of secondary metabolic pathways to produce phenolics, suberin, lignin derivatives, and phytoalexins etc., for antibiosis and antixenosis host-defense strategies [19,41,42,43,44,45]. The reduction in total VOCs also may reflect maladaptation of green ash to EAB, or reduced leaf-to-stem transport of sugars (energy sources for metabolism) due to girdling and phloem damage. In addition, EAB may have a mechanism to repress host defenses, given that both sapwood VOCs and some bark phenolics were reduced in infested trees. Some insects are known to manipulate host defenses in order to improve food quality or living conditions [46,47,48,49].

We identified at least eight VOCs in sapwood emissions from healthy trees that were absent in the sapwood of EAB-infested trees. The compounds were tentatively identified to include aliphatic alcohols, nitriles, aldehydes, esters, ketones, alkanes, and possibly alkyl benzenes. Further research with known chemical standards, MS, and nuclear magnetic resonance (NMR) spectroscopy will be required to confirm VOC identifications. We attribute the broad, strongly-positive response of most sensors composing the smellprint signature of healthy sapwood to be due primarily to these healthy biomarker metabolites. Healthy-plant specific VOCs, considered here as one type of plant-health chemical biomarker (not found in infested trees), may provide an effective means for identifying and chemically detecting green ash trees that have not yet been attacked by EAB. We consider these compounds to be healthy-plant biomarkers of potential utility for early detection of EAB-infestation because these compounds disappear once EAB-infestation occurs. More research will be required to determine if these plant-health biomarker VOCs detected in the sapwood are specific to green ash or also occur in VOC emissions from sapwood of other ash species.

Five unique VOC emissions also were found to be induced only in EAB-infested sapwood. These compounds, identified tentatively to include aliphatic aldehydes, alcohols, salicylate esters, monoterpenes, and sesquiterpenes, were all formed in response to EAB-infestation, but absent from healthy host sapwood. We consider these specific induced VOCs to be EAB-infestation biomarkers, a second type of plant-health chemical biomarker, but indicative of infestations and absent in the sapwood of healthy trees. Following insect damage, major metabolic shifts may occur to seal wound sites, produce defensive compounds, conserve carbon resources, and attract natural enemies (e.g., predatory insects and parasitoids) of invading insect herbivores [50,51,52,53,54]. Presumably these infestation-induced shifts in host metabolic pathways produce different VOCs in infested trees that vicariously replace the more abundant normal VOCs (healthy biomarkers) common to healthy trees. However, the production and release of infestation-induced VOCs into green ash sapwood may or may not be useful or effective in preventing or reducing further gallery formation and expansion. We do not know the source of VOC emissions in the sapwood. Since the high volatility of these compounds allow for their wide diffusion through sapwood tissues from multiple possible sources, they may be produced or released from phloem, cambium, or sapwood tissue itself.

Crook et al. [55] found that manually girdling trees (as a simulation of EAB gallery-formation effects) induced the production of volatile sesquiterpenes, such as α-caryophyllene (α-humulene), β-caryophyllene, α-cubebene, and α-copaene in green ash bark within 24 h. These VOCs consistently elicited EAB antennal responses in both males and females, potentially attracting and bringing them together for mating. It was suggested that mated females use these volatile cues when searching for oviposition sites on bark. Our results indicated the emission of a single constitutive sesquiterpene from green ash sapwood, tentatively identified as isocaryophyllene for peak 13, along with five other constitutive monoterpenes. We also found two induced putative sesquiterpenes in emissions from ash sapwood, identified as β-caryophyllene or other possible isomers for peak 14, and caryophyllene acetate for peak 15. These data provide evidence for additional terpenoids that may be emitted through the bark of ash hosts, both before EAB-infestation (constitutive) and after infestation-induced girdling, which might attract adults and stimulate additional female egg-laying, bole colonization, and gallery formation by larvae.

We detected 192 non-redundant ions by LC-MS in green ash bark methanolic extracts, including some major compounds previously identified in green ash bark, such as verbascoside, ligustroside, and oleuropein. The limited induction of host chemical-defense compounds within bark tissue of EAB larvae-infested bark is consistent with previous studies that found little evidence of constitutive or induced defenses against EAB in North American ash species [26,56,57,58]. However, some previously identified compounds were not abundant enough to detect in our study, such as hydroxytyrosol hexoside, syringin, syringaresinol, caffeoyl-quinic acid, pinoresinol dihexoside, quercetin diglycoside, and apigenin (Table S1). This may indicate intraspecific or regional variations in phenolic chemistry (i.e., ecotypes). However, the majority (118) of organic compounds extracted from green ash bark were not significantly affected by EAB infestations, regardless of decline class (Table S1). Of those compounds that were altered by decline stage, 55 declined in abundance during EAB infestation (Table S2), and only 19 increased (Table S3). Phenolic compounds that were induced to elevated abundance in the bark of EAB-infested trees, still had at least 10-fold lower concentrations than the most abundant phenolic compounds in bark of nonsymptomatic trees. The very limited induction of chemical defenses (host response via increased bark phenolic production) in infested trees is consistent with the results of previous studies that found little evidence of constitutive or induced defenses against EAB in North American ash species, partially explaining the susceptibility of green ash to EAB [26,56,57,58,59,60].

Villari et al. [19] suggested that North American ash species are inherently susceptible to EAB attack because they possess latent defenses that are not induced by larval colonization, perhaps as a result of trees not recognizing larval-infestation cues or responding quickly enough. However, the latent defenses or resistance of some North American species to EAB has been shown to be induced in response to the exogenous application of methyl jasmonate that appears to increase bark concentrations of verbascoside, lignin, and/or trypsin inhibitors in white, green, and black ash, which decreased larval survival and growth [61]. There is a possibility that latent resistance to EAB is cultivar-specific among North American ash species. Several studies have investigated the possibility of some natural host resistance existing in ash trees (referred to as “lingering ash”) surviving two years or more after initial infestation within the northwestern region of Ohio. Of the eleven thousand ash trees surveyed in this region, only approximately 2.6% of these trees have survived and only 1% have retained a healthy crown [62,63]. These trees are being further investigated as potential germplasm sources of EAB genetic resistance in ash-breeding programs. E-nose detection could be useful in such breeding programs to noninvasively screen for the presence of EAB in stems independently of crown health observations.

## 5. Conclusions

Electronic-nose instruments have the potential to be useful devices for early detection of insect infestations of trees based on identification of uniquely specific VOC emissions from affected parts of the host [64,65,66,67]. E-nose devices are capable of recognizing highly specific mixtures of complex VOC metabolites as a single smellprint signature that provides specific information about the metabolic (physiological) health state of the organism (plants, animals, or human) from which gas samples are taken [68,69]. This capability of metabolite-source identification of VOC emissions is possible without having to identify individual chemical species present in the sample mixture [28,70]. This is one of the major strengths of e-nose instruments. It precludes the requirement for complex chemical analysis that can be expensive, yet provides effective discrimination of VOC-metabolite samples from a living host. 

Screening tree sapwood and bark VOC emissions could be used to help facilitate selection of trees for early preemptive and prophylactic EAB-control treatments. EAB adults are attracted to trees that are already infested, which leads to intensified attacks as females continue to lay eggs in the bark of susceptible trees that are already stressed. Consequently, early treatments may help prevent the success of initial EAB attacks, reduce the attraction of infested trees, and slow down or prevent further gallery development and colonization of the tree. We have identified new potential sapwood VOC biomarkers, both specific healthy biomarkers and EAB-infested biomarkers, which may facilitate the identification of uninfested and infested ash trees in the absence of visual EAB-infestation indicators. The use of smellprint signature libraries of green ash trees in various health states could be used to help develop EAB forest pest-management strategies for minimizing established EAB infestations.

Portable e-nose devices and methods could be developed for field-use with application-specific VOC-signature libraries to noninvasively detect healthy and EAB-infestation biomarker emissions from sapwood of individual trees (even at early stages of EAB infestations) to determine their current health status. Such early detections would give land managers a better opportunity to obtain an early indication of the health state of individual trees useful for planning and implementing targeted EAB pest-suppression activities, and to intervene more successfully to protect trees against infestation and subsequent attack through application of early treatments and pest management actions.

## Figures and Tables

**Table 1:** Green ash test-tree and sample characteristics (metadata), and numbers of samples chemically analyzed.

Crown Health Class	Decline Rating ^1^	Trees n=	Mean DBH (cm) ^2^	Mean Tree Height (m) ^3^	Wood Core Samples ^4^	Bark Samples ^4^	Total Samples
Healthy	1	23	18.56	14.28	0.60	0.01	46
Light decline	2	8	15.14	13.02	1.01	0.01	16
Moderate decline	3	4	15.05	11.85	1.06	0.01	8
Severe decline	4	8	14.95	11.97	1.17	0.01	16

^1^ Crown decline rating for green ash trees: 1 = Healthy (no decline or crown dieback), no major branch mortality, no EAB-adult exit holes on bole, no bark splits, no epicormic branches; 2 = light decline (10–25% crown dieback), no EAB-adult exit holes on bole, no bark splits, no epicormic branches; 3 = moderate decline (26–50% crown dieback), EAB-adult exit holes present, bark splits present, and 1–10% epicormics branches; 4 = severe decline (> 50% crown dieback), EAB-adult exit holes present, bark splits present, and > 10% epicormic branches; 5 = dead tree, all leaves, branches, and bark necrotic. EAB; emerald ash borer. DBH; diameter at breast height. ^2^ Mean diameter at breast height (cm). ^3^ Mean total tree height (m). ^4^ Mean dry weight (g) per sample for sapwood core and bark.

**Table 2:** Chemical relatedness between electronic-nose VOC-profiles of green ash tree core headspace volatiles analyzed by 3-d DFA with pattern discrimination index.

Aroma Class 1 ^1^	Aroma Class 2 ^1^	DFA Distance ^2^	PDI (%) ^3^
Healthy (uninfested)	Light infestation	217,953.97	93.06
	Moderate infestation	254,027.19	95.89
	Severe infestation	251,582.30	94.39
Light infestation	Moderate infestation	37,285.54	90.12
	Severe infestation	196,149.00	92.26
Moderate infestation	Severe infestation	217,352.23	92.33

^1^ Green ash sapwood core sample types (aroma classes): Healthy = no EAB-infestation taken from decline rating 1 trees; Light infestation = low EAB-infestation taken from decline rating 2 trees; Moderate infestation = medium-level EAB-infestation taken from decline rating 3 trees; Severe infestation = heavy EAB-infestation taken from decline rating 4 trees. ^2^ DFA distances indicate actual data-plot mapping distances between centers of aroma class data clusters defined by discriminant factor analysis (DFA). ^3^ Pattern discrimination index (PDI) values indicate percentage differences in VOC-metabolite aroma profiles determined by pairwise comparisons of aroma class (sample types) based on DFA tests of aroma signature (smellprint) patterns derived from the e-nose sensor array.

**Table 3:** Metabolomic effects of EAB-infestation on production of constitutive (putative) host VOC-metabolites within green ash sapwood.

			Mean Peak Areas ^1^ by Retention Time (s) ^2^													
		Uniform (Including Outliers) ^3^					Decreasing			Increasing		Uniform (Except Outliers)				
		4 ^4^	5	6	13		2	3		1	9		7	8	11	12
**Canopy Health Class**	**Cores n=**	**21.34 ^2^**	**43.67**	**50.13**	**82.84**	****	**16.43**	**17.91**	****	**13.63**	**58.92**	****	**56.23**	**57.90**	**63.25**	**69.15**
Healthy (uninfested)	23	310 a	160 a	1,503 a	456 a		40,798 b	905 b		452 b	1,820 c		99,115 b	4,691 b	6,141 b	3,066 b
Light decline	6	63 a	140 a	1,381 a	425 a		1,849 b	450 b		546 a	2,330 b		185,732 b	1,712 b	4,898 b	7,992 b
Moderate decline	4	63 a	163 a	1,604 a	449 a		182 c	508 b		586 a	2,280 b		233,210 b	3,083 b	6,521 b	3,846 b
Severe decline	6	69 a	138 a	1,421 a	432 a		140 c	441 c		576 a	2,480 b		107,254 b	1,805 b	5,006 b	2,963 b
Putative—near EAB larval galleries^5^	2	137 a	173 a	1,685 a	414 a		257,113 a	1,523 a		515 a	3,327 a		874,851 a	62,018 a	72,860 a	25,039 a

^1^ Mean peak area (under the curve) for chromatographic peaks (numbered) of each individual VOC-metabolite (at the indicated retention time). Values followed by the same letter in each column are not significantly different according to Brown–Forsythe Equal Variance test and Fisher LSD-tests at (P < 0.05). ^2^ Retention time (to 0.01 s) of VOC-metabolite in DB-5 column by gas chromatography. ^3^ Category of metabolomic changes (uniform, decreasing, or increasing) of constitutive sapwood VOC-emissions in declining green ash trees (relative to healthy, uninfested trees). ^4^ Peak number within gas chromatogram; see corresponding peak numbers in Table 5, giving tentative identities of VOCs for each peak. ^5^ Tree cores taken from among trees with severe EAB-decline rating which we putatively believe were located very close to EAB larval galleries due to exceptionally high (outlier) GC-peak areas for some VOC-metabolites recorded for these cores.

**Table 4:** Aroma class-specific production of VOC-metabolites by healthy host and EAB-decline associated response within green ash sapwood.

		Mean Peak Areas ^1^ by Retention Time (s) ^2^																
		Healthy Host (Non-Decline) ^3^									Nonspecific			EAB-Decline (Induced/Insect)				
		1 ^4^	2	4	5	6	8	12	13		3	7		9	10	11	14	15
**Crown Health Class**	**Cores n=**	**15.35 ^2^**	**19.18**	**25.85**	**26.79**	**29.98**	**42.06**	**79.78**	**80.48**	****	**23.41**	**38.93**	****	**72.88**	**74.01**	**74.98**	**86.32**	**96.80**
Healthy (uninfested)	23	804	1,605	5,038	1,803	212	503	265	155		593	279		T	–	T	–	–
Light decline	6	–	–	–	–	–	–	–	–		299	470		–	114	–	77	71
Moderate decline	4	–	–	–	–	–	–	–	–		–	230		87	137	–	60	354
Severe decline	6	–	–	–	–	–	–	–	–		221	102		–	100	–	85	113
Putative—near EAB larval galleries ^5^	2	–	–	–	–	–	–	–	–		2,173	4,579		830	97	250	96	81

^1^ Mean peak area (under the curve) for chromatographic peaks (numbered) of each individual VOC-metabolite (at the indicated retention time). ^2^ Retention time (to 0.01 sec) of VOC-metabolite in DB-5 column by gas chromatography; **–** = not produced; T = trace amount rarely produced. ^3^ Category of likely source and occurrence (healthy host, nonspecific source, EAB decline-induced in host or of insect origin) of sapwood VOC-emissions in green ash trees. ^4^ Peak number within gas chromatogram; see corresponding peak numbers in Table 6, giving tentative identities of VOCs for each peak. ^5^ Tree cores taken from among trees with severe EAB-decline rating, which we putatively believe were located very close to EAB larval galleries due to exceptionally high GC-peak areas for VOC-metabolites recorded for these cores.

**Table 5:** Gas chromatographic data indicating tentative identities of constitutive VOC-metabolite emissions derived from host sapwood of green ash.

Peak	RT ^1^	KRI-v ^2^	Tentative Identity	CAS No. ^3^	KRI-t ^4^	RI Range ^5^	Chemical Class
1	13.63	411	Methyl formate	107-31-3	401	66.19-85.78	Methyl ester
2	16.43	474	Propanal	123-38-6	499	48.02-73.06	Aliphatic aldehyde
			Propane-2-one	67-64-1	498	61.93-66.03	Aliphatic ketone
3	17.91	508	Pentane	109-66-0	500	30.02-78.35	Alkane
4	21.34	587	2,3-butanedione	431-03-8	589	74.13-93.26	Aliphatic diketone
			Vinyl acetate	108-05-4	582	56.26-92.16	Aliphatic ester
5	43.67	814	Butanoic acid	107-92-6	812	86.22-94.05	Carboxylic acid
			E-2-Octene	13389-42-9	815	51.56-94.27	Alkene
6	50.13	877	3-mercapto-4-methyl-2-pentanone	75832-79-0	883	39.83-86.66	Sulfhydryl ketone
			Dimethyl methylphosphonate	756-79-6	880	47.21-86.53	Phosphonic dimethyl ester
7	56.23	948	1 S-(-)-α-pinene	7785-26-4	943	44.58-90.61	Bicyclic monoterpene
			4-methylnonane	17301-94-9	962	40.95-85.12	Alkane
8	57.90	966	3-ethyloctane	5881-17-4	969	76.22-96.76	Alkane
			Amyl-propanoate	624-54-4	972	83.21-95.67	Aliphatic ester
			3-methylnonane	5911-04-6	971	75.25-94.76	Alkane
9	58.92	981	β-pinene	127-91-3	979	83.35-91.94	Bicyclic monoterpene
			Phenol	108-95-2	986	81.21-91.63	Aromatic hydroxide
10	59.88	994	Hexanoic acid	142-62-1	996	86.28-97.16	Carboxylic acid
			1,3,5-trimethyl-benzene	108-67-8	994	85.59-92.29	Benzene deriv.
11	63.25	1042	Limonene	138-86-3	1049	71.49-89.57	Cyclic monoterpene
			1,8-cineole	470-82-6	1040	63.29-81.97	Bicyclic monoterpene
12	69.15	1135	Ethyl cyclohexane-carboxylate	3289-28-9	1136	93.87	Homocyclic ethyl ester
			Cis-rose oxide	3033-23-6	1127	81.41-88.33	Pyran monoterpene
13	82.84	1405	Methyl eugenol	93-15-2	1404	63.18-96.25	Benzene diester
			δ-nonalactone	3301-94-8	1404	58.74-76.73	Lactone deriv.
			isocaryophyllene	118-65-0	1405	70.25	Bicyclic sesquiterpene

^1^ Retention times (to 0.01 s) for VOC-metabolites derived from headspace of green ash sapwood core samples run within a 10 m DB-5 column using GC-analysis parameters specified previously. ^2^ KRI-v = Kovats Retention Index for specific volatile metabolite represented by the individual peak and retention time when with a 10 m DB-5 column using 11-alkane (C7-C17) analytical reference standard calibration. ^3^ CAS number = Chemical Abstracts Service (CAS) Registry Number, unique numerical identifier. ^4^ KRI-t = Kovats Retention Index for tentative identify for compounds; indicated as most probable identity based on closest KRI-values. ^5^ RI = Relevance Index, indicating percentage probability of identity match, based on Kovats values for the specified tentative-identity reference compounds, determined from dual-column data derived from 10 m DB-5 and DB-1701 columns with analytical reference standards.

**Table 6:** Gas chromatographic data indicating tentative identities of healthy host and induced EAB-decline associated response VOC-metabolite emissions from sapwood of green ash.

Peak	RT ^1^	KRI-v ^2^	Tentative Identity	CAS No. ^3^	KRI-t ^4^	RI Range ^5^	Chemical Class
1	15.35	451	Ethanol	64-17-5	449	74.17–94.19	Aliphatic alcohol
			Methanethiol	74-93-1	448	83.82–90.25	Aliphatic thiol
2	19.18	538	Acetonitrile	75-05-8	539	81.26–89.55	Nitrile deriv.
			Acrylonitrile	107-13-1	526	76.47–84.35	Nitrile deriv.
3	23.41	617	1-propanethiol	107-03-9	616	49.83–88.24	Aliphatic thiol
			Acetic acid	64-19-7	619	84.70	Carboxylic acid
4	25.85	646	3-methylbutanal	590-86-3	652	53.26–66.95	Aliphatic aldehyde
			(E)-but-2-enal	15798-64-8	646	53.63–67.32	Aliphatic aldehyde
5	26.79	656	Isopropyl acetate	108-21-4	650	57.63–80.47	Aliphatic ester
			1-hydroxy-2-propanone	116-09-6	654	52.60–73.57	Aliphatic ketone
6	29.98	693	2-pentanone	107-87-9	688	89.71–94.23	Aliphatic ketone
			Pentanal	110-62-3	698	84.35–93.87	Aliphatic aldehyde
			2,3-pentanedione	600-14-6	698	93.76	Aliphatic diketone
7	38.93	772	2-methylthiophene	554-14-3	775	90.18–91.90	Heterocyclic thiol
			Prenol	556-82-1	775	52.43–91.43	Alcohol terpenoid
8	42.06	799	Hexanal	66-25-1	801	85.84–95.35	Aliphatic aldehyde
9	72.88	1201	6-decenal	127818-71-7	1203	81.11–97.35	Aliphatic aldehyde
			2-decanol	1120-06-5	1200	93.58–93.63	Aliphatic alcohol
			methyl salicylate	119-36-8	1214	76.16	Aromatic ester
10	74.01	1223	Nerol	106-25-2	1228	74.10–94.13	Monoterpene
			3-decen-2-one	10519-33-2	1233	85.17–89.13	Aliphatic ketone
			2-[(methyldithio) methyl] furan	57500-00-2	1226	88.97	Dithio furan
			Quinoxaline	91-19-0	1229	73.42–86.85	Benzopyrazine
11	74.98	1242	hexyl 2-butenoate	19089-92-0	1238	82.89	Aliphatic ester
12	79.78	1335	Heptyl crotonate	16930-99-7	1335	52.54–88.79	Heptyl ester
			Heptylcyclohexane	5617-41-4	1346	65.59–88.75	Cyclohexane deriv.
13	80.48	1338	7-methyl-tridecane	26730-14-3	1351	80.69–87.57	Alkane deriv.
			Heptylbenzene	1078-71-3	1368	60.02–73.14	Alkylbenzene
14	86.32	1482	β-caryophyllene	87-44-5	1482	84.09–91.61	Bicyclic sesquiterpene
			cis-caryophyllene	118-65-0	1482	NA	Bicyclic sesquiterpene
			9-epi-caryophyllene	unspecified	1467	NA	Bicyclic sesquiterpene
			Butyl salicylate	2052-14-4	1468	NA	Aromatic ester
15	96.80	1728	α-bisabolol oxide A	22567-36-8	1744	NA	Monocyclic sesquiterpene alcohol
			Caryophyllene acetate	57082-24-3	1704	NA	Bicyclic sesquiterpene ester

^1^ Retention times (to 0.01 sec) for VOC-metabolites derived from headspace of green ash sapwood core samples run within a 10 m DB-5 column using GC-analysis parameters specified previously. ^2^ KRI-v = Kovats Retention Index for specific volatile metabolite represented by the individual peak and retention time for a 10 m DB-5 column using 11-alkane (C7-C17) analytical reference-standard calibration. ^3^ CAS number = Chemical Abstracts Service (CAS) Registry Number, unique numerical identifier. ^4^ KRI-t = Kovats Retention Index for tentative identify for compounds; indicated as most probable identity based on closest KRI-values. ^5^ RI = Relevance Index, indicating percentage probability of identity match, based on Kovats values for the specified tentative-identity reference compounds, determined from dual-column data derived from 10 m DB-5 and DB-1701 columns with analytical reference standards; NA = not available (due to limited data from all samples).

**Table 7:** Relative presence of the sixteen most abundant phenolic compounds detected in green ash bark of trees from different classes of EAB-decline.

						LC-MS Signal Intensity (×10^6^) EAB Decline Class					Fold Difference				P= ^6^		
Peak	RT ^1^	m/z ^2^	Fragments ^3^	UVmax ^4^	Tentative ID ^5^	1	2	3	4		2:1	3:1	4:1		1 v 2	1 v 3	1 v 4
9	1.3	341.08	179, 161, 143	NA	Unidentified	1.2 ± 0.3	1.1 ± 0.1	1.2 ± 0.2	0.9 ± 0.2		0.89	0.94	**0.75**		0.30	0.65	**0.03**
12	1.4	711.17	665	NA	Unidentified	5.0 ± 1.6	3.7 ± 1.4	2.6 ± 1.6	1.3 ± 1.1		0.74	**0.51**	**0.27**		0.07	**0.01**	**< 0.01**
16	4.6	461.25	315, 135, 297	270	Unidentified	1.0 ± 0.3	1.0 ± 0.1	1.3 ± 0.3	1.1 ± 0.4		1.00	**1.37**	1.16		0.99	**0.05**	0.27
20	5.2	431, 477*	299, 149, 191	276	Unidentified	8.9 ± 1.4	8.5 ± 1.3	8.8 ± 2.2	8.2 ± 1.6		0.95	0.98	0.92		0.47	0.87	0.26
24	5.3	431,477*	431	303, 267	Unidentified	1.8 ± 0.3	1.7 ± 0.2	1.8 ± 0.4	1.6 ± 0.3		0.92	0.96	0.88		0.26	0.67	0.08
28	5.5	417	209, 207, 371	265	Unidentified	1.8 ± 0.3	1.5 ± 0.3	1.6 ± 0.2	1.5 ± 0.2		**0.84**	0.89	0.87		**0.04**	0.25	0.08
58	8.6	581.2	535, 373	279	Unidentified	2.0 ± 0.6	1.4 ± 0.7	2.1 ± 0.7	1.3 ± 0.3		**0.70**	1.07	**0.65**		**0.04**	0.69	**< 0.01**
66	9.2	521, 567*	341, 329, 521	281	Unidentified	1.6 ± 0.3	1.3 ± 0.3	1.5 ± 0.3	1.8 ± 0.6		**0.84**	0.99	1.15		**0.04**	0.89	0.16
72	9.9	623.33	**461**	330, sh-291	Verbascoside	10.1 ± 3.2	7.2 ± 2.9	9.3 ± 3.7	7.8 ± 1.8		**0.71**	0.92	0.77		**0.04**	0.64	0.08
88	10.8	623.42	**461**	327, sh-290	Verbascoside	15.7 ± 2.7	13.4 ± 2.0	16.6 ± 3.1	14.9 ± 2.1		**0.86**	1.06	0.95		**0.05**	0.56	0.48
103	11.5	539.25	377, 275, 359	275^x^	Oleuropein	7.6 ± 3.4	5.1 ± 1.4	5.7 ± 2.1	5.0 ± 0.5		0.66	0.74	**0.66**		0.06	0.28	**0.05**
107	11.7	577, 623*	577, **461**, 415	280	Verbascoside	1.5 ± 1.2	0.7 ± 0.6	0.4 ± 0.3	1.3 ± 1.2		0.46	0.25	0.84		0.09	0.07	0.65
127	12.8	637.33	**461**, 491, 475	326, sh-297	Verbascoside	1.8 ± 0.7	1.8 ± 0.3	2.0 ± 0.6	1.9 ± 0.5		0.98	1.09	1.02		0.86	0.66	0.92
139	14.1	851.25	689	220, 294	Unidentified	2.1 ± 0.5	2.0±0.5	2.2±0.1	2.3±0.6		0.92	1.01	1.06		0.38	0.95	0.57
149	14.9	523, 569*	361, 291, 259	sh-279	Ligustroside	12.6 ± 3.2	8.5 ± 1.7	12.7 ± 2.6	10.7 ± 1.6		**0.67**	1.00	0.85		**< 0.01**	0.97	0.13
154	15.7	701.00	327, 283, 507	326, sh-296	Oleuropein hexoside	1.7 ± 0.8	1.3 ± 0.6	1.3 ± 0.6	2.1 ± 1.1		0.79	0.79	1.25		0.31	0.42	0.28

^1^ Retention time (to 0.1 min) of bark metabolites in a 10 cm × 2.1 mm-id Accucore Vanquish C18+ column, 1.5 µm particle size, maintained at 35 °C with column oven. ^2^ Deprotonated parent [M-H]**^–^** ion mass (*m/z*); * = formide adducts; the most abundant ions were compiled for each EAB-decline class; redundant ions, formide adducts, and electrospray-induced dimers were removed from the list. ^3^ Mass spectrum *m/z* of fragment ions (top 3, ordered by decreasing intensity). ^4^ Wavelength (λ in nm) within UV spectrum (of each compound) with maximum peak absorbance. ^5^ Tentative identification of possible isomeric forms of bark compounds based on retention time, mass spectral and UV spectral data consistent with literature; ligustroside and Oleuropein isomers are in the secoiridoid glycoside chemical subclass; verbascoside isomers are in the phenyl ethanoid chemical subclass. ^6^ P-values (probability of differences) in comparisons between compound abundance in bark of the four EAB-decline classes (1–4) based on Student’s t-test statistical analyses. Values in bold are significantly different at P ≤ 0.05; Values with colored highlights indicate: Cyan = significantly lower compound abundance, green = significantly higher compound abundance.

## Supplementary Materials

The following are available online at https://www.mdpi.com/2079-6374/9/4/123/s1, Table S1: Green ash bark methanol-extractable compounds that have been previously identified. Table S2: Compounds significantly decreased during EAB infestation with peak areas from LC-MS analysis of green ash bark methanolic extracts. Table S3: LC-MS peak areas for green ash bark methanol-extractable contents that were significantly increased by EAB infestation.
