# Supplementary material for: Detection of Emerald Ash Borer Infestations in Living Green Ash by Noninvasive Electronic-Nose Analysis of Wood Volatiles
Source: Biosensors (Basel). 2019 Oct 13;9(4):123. doi: 10.3390/bios9040123 (PMC6956047; doi:10.3390/bios9040123)
Supplement: Supplementary file 1 [file biosensors-09-00123-s001.zip › biosensors-608360-supplementary/Table S1 Supplemental materials - LC-MS 1.docx]

| **Table S1.** Green ash bark methanol-extractable compounds that have been previously identified. | | | | | | | | | | | | | | | | | | | | | | | | | | |
| --- | --- | --- | --- | --- | --- | --- | --- | --- | --- | --- | --- | --- | --- | --- | --- | --- | --- | --- | --- | --- | --- | --- | --- | --- | --- | --- |
|  |  |  |  |  |  |  | Decline class (LCMS signal intensity x 100,000) | | | | | | | | | | | |  | Fold difference | | |  | P | | |
| No. | RT | m/z | MS2 | UV max | Tentative ID |  | 1 | | | 2 | | | 3 | | | 4 | | |  | 2 | 3 | 4 |  | 1 v 2 | 1 v 3 | 1 v 4 |
| 18 | 4.75 | 345*, 299 | 179, 119, 143, 161, 113 | 276 | Tyrosol hexoside |  | 2.9 | ± | 1.2 | 1.8 | ± | 0.8 | 2.6 | ± | 0.9 | 2.5 | ± | 0.5 |  | **0.61** | 0.92 | 0.86 |  | **0.03** | 0.71 | 0.37 |
| 20 | 5.18 | 431, 477* | 299, 149, 191, 131, 251 | 276 | Unknown |  | 89.5 | ± | 14.1 | 85.1 | ± | 12.8 | 88.1 | ± | 22.1 | 82.3 | ± | 15.8 |  | 0.95 | 0.98 | 0.92 |  | 0.47 | 0.87 | 0.26 |
| 45 | 7.48 | 785 | 623 | 332, sh 292 | Forsythoside A *O*-glucoside |  | 10.3 | ± | 10.4 | 5.0 | ± | 2.0 | 10.9 | ± | 2.9 | 6.2 | ± | 3.4 |  | **0.48** | 1.06 | **0.61** |  | 0.20 | 0.91 | 0.32 |
| 52 | 8.3 | 523, 569* | 361 | 277 | Ligustroside |  | 2.9 | ± | 0.9 | 2.0 | ± | 1.4 | 2.8 | ± | 0.4 | 2.8 | ± | 1.6 |  | **0.67** | 0.94 | 0.96 |  | **0.05** | 0.73 | 0.82 |
| 55 | 8.5 | 581*, 535 | 373, 489 | 278 | Hydroxypinoresinol hexoside |  | 2.8 | ± | 0.9 | 2.0 | ± | 0.9 | 3.4 | ± | 1.1 | 2.3 | ± | 0.6 |  | **0.72** | 1.23 | 0.84 |  | 0.06 | 0.23 | 0.24 |
| 72 | 9.87 | 623.33 | 461 | 330, sh 291 | Verbascoside |  | 101.3 | ± | 32.0 | 72.3 | ± | 29.0 | 92.9 | ± | 37.2 | 78.2 | ± | 18.2 |  | **0.71** | 0.92 | 0.77 |  | **0.04** | 0.64 | 0.08 |
| 82 | 10.55 | 565*, 519 | 357 | 278 | Pinoresinol glucoside |  | 1.8 | ± | 0.9 | 1.1 | ± | 0.5 | 2.6 | ± | 0.5 | 1.9 | ± | 1.1 |  | **0.61** | **1.40** | 1.06 |  | **0.05** | 0.12 | 0.78 |
| 88 | 10.75 | 623.42 | 461 | 327, sh 290 | Verbascoside |  | 156.8 | ± | 27.4 | 134.2 | ± | 19.9 | 165.7 | ± | 31.2 | 148.8 | ± | 21.4 |  | 0.86 | 1.06 | 0.95 |  | 0.05 | 0.56 | 0.48 |
| 97 | 11.27 | 731*, 685 | 299, 523, 223, 453, 421 | 308 | Nuzhenide |  | 0.8 | ± | 0.4 | 0.4 | ± | 0.1 | 0.4 | ± | 0.1 | 0.4 | ± | 0.1 |  | **0.53** | **0.56** | **0.46** |  | **0.04** | 0.14 | **0.02** |
| 103 | 11.45 | 539.25 | 377, 275, 359, 291 | 275^§^ | Oleuropein |  | 76.4 | ± | 33.8 | 50.5 | ± | 14.4 | 56.7 | ± | 21.1 | 50.1 | ± | 5.5 |  | **0.66** | **0.74** | **0.66** |  | 0.06 | 0.28 | 0.05 |
| 107 | 11.7 | 623*, 577 | 577, 461, 415 (577 MS2: 415) | 280 | Verbascoside-related |  | 14.9 | ± | 11.6 | 6.8 | ± | 6.4 | 3.7 | ± | 2.5 | 12.5 | ± | 12.4 |  | **0.46** | **0.25** | 0.84 |  | 0.09 | 0.07 | 0.65 |
| 128 | 12.83 | 539, 585* | 377, 275, 359, 291 | sh 274 | Oleuropein |  | 1.0 | ± | 0.9 | 0.8 | ± | 0.7 | 0.6 | ± | 0.2 | 1.1 | ± | 1.4 |  | 0.80 | **0.60** | 1.10 |  | 0.59 | 0.39 | 0.82 |
| 149 | 14.87 | 523, 569* | 361, 291, 259 | sh 279 | Ligustroside |  | 126.0 | ± | 31.9 | 84.6 | ± | 16.5 | 126.5 | ± | 26.3 | 106.5 | ± | 15.8 |  | **0.67** | 1.00 | 0.85 |  | **<0.01** | 0.97 | 0.13 |
| 154 | 15.66 | 701.00 | 327, 283, 507, 495, 261, 539, 301, 345 | 326, sh 296 | Oleuropein hexoside(?) |  | 17.0 | ± | 8.3 | 13.5 | ± | 6.0 | 13.4 | ± | 5.7 | 21.3 | ± | 11.5 |  | 0.79 | 0.79 | **1.25** |  | 0.31 | 0.42 | 0.28 |
| 160 | 17.25 | 701 | 539, 507, 463, 437, 359 | NA | Oleuropein hexoside(?) |  | 0.9 | ± | 0.6 | 0.6 | ± | 0.6 | 1.6 | ± | 0.7 | 0.7 | ± | 0.6 |  | **0.68** | **1.85** | 0.82 |  | 0.31 | **0.04** | 0.56 |
|  |  | * formide adduct | |  |  |  |  |  |  |  |  |  |  |  |  |  |  |  |  |  |  |  |  |  |  |  |

Compounds included here were identified in green ash in a previous report (Eyles et al. 2007; Whitehill et al., 2012). Statistical significance is indicated by bold orange, α = 0.05, or bold light orange, α < 0.01. Peak areas that were 25% greater than for healthy trees are indicated by bold red; light red indicates at least 50% higher peak area; blue font indicates at least 25% higher peak area; light blue indicates at least 50% lower peak area.
