# Supplementary material for: Detection of Emerald Ash Borer Infestations in Living Green Ash by Noninvasive Electronic-Nose Analysis of Wood Volatiles
Source: Biosensors (Basel). 2019 Oct 13;9(4):123. doi: 10.3390/bios9040123 (PMC6956047; doi:10.3390/bios9040123)
Supplement: Supplementary file 1 [file biosensors-09-00123-s001.zip › biosensors-608360-supplementary/Table S2 Supplemental materials - LC-MS 2.docx]

| **Table S2.** Compounds significantly decreased during EAB infestation with peak areas from LCMS analysis of green ash bark methanolic extracts. | | | | | | | | | | | | | | | | | | | | | | | |
| --- | --- | --- | --- | --- | --- | --- | --- | --- | --- | --- | --- | --- | --- | --- | --- | --- | --- | --- | --- | --- | --- | --- | --- |
|  |  |  |  | Decline class (LCMS signal intensity x 10^5^) | | | | | | | | | | | |  | Fold difference | | |  | P | | |
| **No.** | **RT** | **m/z** |  | 1 | | | 2 | | | 3 | | | 4 | | |  | 2 | 3 | 4 |  | 1 v 2 | 1 v 3 | 1 v 4 |
| 146 | 14.8 | 569 |  | 43.5 | ± | 10.6 | 29.2 | ± | 4.4 | 44.1 | ± | 9.1 | 35.6 | ± | 5.9 |  | **0.67** | 1.02 | 0.82 |  | **< 0.01** | 0.91 | 0.08 |
| 149 | 14.87 | 523 |  | 126.0 | ± | 31.9 | 84.6 | ± | 16.5 | 126.5 | ± | 26.3 | 106.5 | ± | 15.8 |  | **0.67** | 1.00 | 0.85 |  | **< 0.01** | 0.97 | 0.13 |
| 145 | 14.75 | 291 |  | 9.4 | ± | 2.5 | 6.3 | ± | 1.2 | 9.3 | ± | 1.8 | 7.9 | ± | 1.3 |  | **0.66** | 0.98 | 0.83 |  | **< 0.01** | 0.90 | 0.13 |
| 144 | 14.72 | 361 |  | 16.0 | ± | 4.1 | 10.8 | ± | 2.0 | 15.8 | ± | 3.6 | 13.7 | ± | 2.3 |  | **0.68** | 0.99 | 0.86 |  | **< 0.01** | 0.93 | 0.17 |
| 147 | 14.8 | 1047 |  | 40.4 | ± | 15.0 | 21.5 | ± | 8.0 | 40.7 | ± | 12.1 | 30.3 | ± | 7.0 |  | **0.53** | 1.01 | **0.75** |  | **< 0.01** | 0.98 | 0.10 |
| 65 | 9.09 | 565 |  | 4.1 | ± | 0.9 | 2.9 | ± | 0.8 | 3.4 | ± | 0.3 | 3.4 | ± | 1.1 |  | **0.71** | 0.83 | 0.83 |  | **< 0.01** | 0.14 | 0.09 |
| 36 | 6.38 | 583 |  | 5.3 | ± | 1.3 | 3.4 | ± | 1.4 | 3.7 | ± | 0.8 | 5.3 | ± | 2.2 |  | **0.65** | **0.70** | 1.01 |  | **< 0.01** | **0.03** | 0.92 |
| 46 | 7.59 | 757 |  | 2.9 | ± | 0.8 | 1.9 | ± | 0.8 | 2.1 | ± | 0.5 | 2.5 | ± | 0.9 |  | **0.67** | **0.75** | 0.89 |  | **< 0.01** | 0.09 | 0.36 |
| 35 | 6.35 | 403 |  | 1.8 | ± | 0.6 | 1.2 | ± | 0.3 | 1.3 | ± | 0.3 | 1.0 | ± | 0.3 |  | **0.66** | **0.70** | **0.56** |  | **0.01** | 0.09 | **< 0.01** |
| 141 | 14.27 | 523 |  | 1.1 | ± | 0.4 | 0.7 | ± | 0.2 | 1.3 | ± | 0.4 | 1.3 | ± | 0.4 |  | **0.65** | 1.20 | 1.23 |  | **0.02** | 0.31 | 0.13 |
| 63 | 9.07 | 521 |  | 2.1 | ± | 1.1 | 1.0 | ± | 0.3 | 2.3 | ± | 1.2 | 3.8 | ± | 2.7 |  | **0.50** | 1.09 | **1.82** |  | **0.02** | 0.75 | **0.02** |
| 37 | 6.54 | 583 |  | 5.0 | ± | 1.1 | 3.7 | ± | 1.6 | 3.8 | ± | 0.9 | 4.8 | ± | 1.9 |  | **0.74** | 0.75 | 0.97 |  | **0.02** | 0.05 | 0.77 |
| 133 | 13.58 | 569 |  | 5.5 | ± | 2.5 | 3.0 | ± | 1.5 | 2.8 | ± | 2.2 | 1.9 | ± | 0.7 |  | **0.55** | **0.52** | **0.34** |  | **0.02** | 0.06 | **< 0.01** |
| 17 | 4.74 | 345 |  | 7.9 | ± | 3.2 | 4.8 | ± | 2.3 | 7.2 | ± | 2.4 | 6.5 | ± | 1.6 |  | **0.61** | 0.91 | 0.83 |  | **0.03** | 0.69 | 0.29 |
| 8 | 1.3 | 1053 |  | 5.9 | ± | 1.9 | 4.0 | ± | 1.5 | 3.0 | ± | 2.1 | 1.3 | ± | 1.1 |  | **0.69** | **0.52** | **0.23** |  | **0.03** | **0.01** | **< 0.01** |
| 18 | 4.75 | 299 |  | 2.9 | ± | 1.2 | 1.8 | ± | 0.8 | 2.6 | ± | 0.9 | 2.5 | ± | 0.5 |  | **0.61** | 0.92 | 0.86 |  | **0.03** | 0.71 | 0.37 |
| 26 | 5.39 | 611 |  | 8.3 | ± | 3.8 | 5.0 | ± | 1.1 | 5.3 | ± | 2.0 | 4.8 | ± | 2.2 |  | **0.59** | **0.64** | **0.57** |  | **0.03** | 0.13 | **0.03** |
| 30 | 5.5 | 611 |  | 8.3 | ± | 3.7 | 4.9 | ± | 1.1 | 5.3 | ± | 1.9 | 4.7 | ± | 2.2 |  | **0.59** | **0.64** | **0.57** |  | **0.03** | 0.13 | **0.02** |
| 29 | 5.5 | 403 |  | 1.4 | ± | 0.6 | 0.9 | ± | 0.2 | 0.9 | ± | 0.4 | 0.8 | ± | 0.3 |  | **0.61** | **0.64** | **0.54** |  | **0.03** | 0.14 | **0.02** |
| 47 | 7.89 | 785 |  | 2.4 | ± | 1.8 | 0.9 | ± | 0.8 | 0.6 | ± | 0.2 | 3.6 | ± | 5.1 |  | **0.36** | **0.25** | **1.45** |  | **0.04** | 0.06 | 0.38 |
| 81 | 10.57 | 357 |  | 1.1 | ± | 0.5 | 0.7 | ± | 0.3 | 1.6 | ± | 0.3 | 1.2 | ± | 0.7 |  | **0.60** | **1.39** | 1.03 |  | **0.04** | 0.11 | 0.87 |
| 80 | 10.5 | 567 |  | 0.3 | ± | 0.2 | 0.2 | ± | 0.1 | 0.5 | ± | 0.1 | 0.4 | ± | 0.2 |  | **0.61** | **1.37** | 1.07 |  | **0.04** | 0.13 | 0.73 |
| 83 | 10.56 | 565 |  | 5.3 | ± | 2.4 | 3.2 | ± | 1.5 | 7.4 | ± | 1.4 | 5.7 | ± | 3.3 |  | **0.61** | **1.38** | 1.07 |  | **0.04** | 0.11 | 0.73 |
| 58 | 8.64 | 581 |  | 19.9 | ± | 6.3 | 13.9 | ± | 7.2 | 21.3 | ± | 6.7 | 12.8 | ± | 3.4 |  | **0.70** | 1.07 | **0.65** |  | **0.04** | 0.69 | **< 0.01** |
| 123 | 12.42 | 527 |  | 2.9 | ± | 1.2 | 1.9 | ± | 0.8 | 3.4 | ± | 0.8 | 3.3 | ± | 0.7 |  | **0.63** | 1.14 | 1.11 |  | **0.04** | 0.53 | 0.53 |
| 99 | 11.27 | 731 |  | 4.2 | ± | 2.9 | 1.8 | ± | 0.8 | 1.9 | ± | 0.6 | 1.5 | ± | 1.0 |  | **0.43** | **0.45** | **0.35** |  | **0.04** | 0.12 | **0.02** |
| 102 | 11.42 | 1079 |  | 31.3 | ± | 20.4 | 14.3 | ± | 6.8 | 17.7 | ± | 12.3 | 13.6 | ± | 2.6 |  | **0.46** | **0.57** | **0.44** |  | **0.04** | 0.21 | **0.03** |
| 72 | 9.87 | 623 |  | 101.3 | ± | 32.0 | 72.3 | ± | 29.0 | 92.9 | ± | 37.2 | 78.2 | ± | 18.2 |  | **0.71** | 0.92 | 0.77 |  | **0.04** | 0.64 | 0.08 |
| 97 | 11.27 | 685 |  | 0.8 | ± | 0.4 | 0.4 | ± | 0.1 | 0.4 | ± | 0.1 | 0.4 | ± | 0.1 |  | **0.53** | **0.56** | **0.46** |  | **0.04** | 0.14 | **0.02** |
| 33 | 6.23 | 583 |  | 3.5 | ± | 1.7 | 2.0 | ± | 1.2 | 3.6 | ± | 2.2 | 2.9 | ± | 1.3 |  | **0.59** | 1.03 | 0.83 |  | **0.05** | 0.92 | 0.39 |
| 104 | 11.48 | 291 |  | 1.7 | ± | 0.7 | 1.1 | ± | 0.3 | 1.2 | ± | 0.5 | 1.1 | ± | 0.2 |  | **0.65** | **0.73** | **0.64** |  | **0.05** | 0.25 | **0.04** |
| 52 | 8.3 | 523 |  | 2.9 | ± | 0.9 | 2.0 | ± | 1.4 | 2.8 | ± | 0.4 | 2.8 | ± | 1.6 |  | **0.67** | 0.94 | 0.96 |  | **0.05** | 0.73 | 0.82 |
| 82 | 10.55 | 519 |  | 1.8 | ± | 0.9 | 1.1 | ± | 0.5 | 2.6 | ± | 0.5 | 1.9 | ± | 1.1 |  | **0.61** | **1.40** | 1.06 |  | **0.05** | 0.12 | 0.78 |
| 136 | 13.94 | 523 |  | 0.9 | ± | 0.3 | 0.6 | ± | 0.2 | 1.2 | ± | 0.3 | 1.1 | ± | 0.3 |  | **0.69** | **1.28** | 1.22 |  | **0.05** | 0.17 | 0.16 |
| 31 | 5.54 | 789 |  | 3.1 | ± | 0.8 | 2.3 | ± | 1.2 | 2.7 | ± | 0.5 | 2.6 | ± | 0.5 |  | **0.74** | 0.85 | 0.82 |  | **0.05** | 0.28 | 0.08 |
| 105 | 11.48 | 585 |  | 32.8 | ± | 14.0 | 21.8 | ± | 5.4 | 24.0 | ± | 8.7 | 21.6 | ± | 2.9 |  | **0.67** | **0.73** | **0.66** |  | 0.06 | 0.24 | **0.05** |
| 7 | 1.3 | 701 |  | 2.4 | ± | 0.9 | 1.7 | ± | 0.6 | 1.4 | ± | 0.8 | 0.8 | ± | 0.6 |  | **0.69** | **0.59** | **0.33** |  | 0.06 | 0.05 | **< 0.01** |
| 113 | 12.12 | 637 |  | 2.4 | ± | 0.8 | 1.8 | ± | 0.8 | 2.1 | ± | 0.7 | 1.8 | ± | 0.4 |  | **0.72** | 0.86 | **0.73** |  | 0.06 | 0.43 | **0.05** |
| 6 | 1.3 | 549 |  | 7.0 | ± | 1.6 | 5.7 | ± | 1.1 | 4.9 | ± | 2.0 | 3.3 | ± | 1.9 |  | 0.82 | **0.70** | **0.47** |  | 0.06 | **0.03** | **< 0.01** |
| 56 | 8.64 | 373 |  | 2.9 | ± | 0.9 | 2.0 | ± | 1.1 | 3.0 | ± | 1.0 | 1.9 | ± | 0.5 |  | **0.71** | 1.06 | **0.67** |  | 0.06 | 0.76 | **0.02** |
| 3 | 1.26 | 665 |  | 12.5 | ± | 4.0 | 9.2 | ± | 3.7 | 6.3 | ± | 4.0 | 3.4 | ± | 2.8 |  | **0.73** | **0.51** | **0.27** |  | 0.06 | **< 0.01** | **< 0.01** |
| 59 | 8.64 | 1117 |  | 1.8 | ± | 1.0 | 1.0 | ± | 0.8 | 2.2 | ± | 1.2 | 0.8 | ± | 0.4 |  | **0.54** | 1.17 | **0.44** |  | 0.06 | 0.60 | **0.02** |
| 12 | 1.35 | 711 |  | 50.3 | ± | 16.5 | 37.1 | ± | 14.1 | 25.9 | ± | 16.2 | 13.4 | ± | 11.5 |  | **0.74** | **0.51** | **0.27** |  | 0.07 | **0.01** | **< 0.01** |
| 4 | 1.27 | 1377 |  | 4.0 | ± | 2.1 | 2.4 | ± | 1.6 | 1.3 | ± | 1.1 | 0.5 | ± | 0.6 |  | **0.60** | **0.33** | **0.11** |  | 0.07 | **0.02** | **< 0.01** |
| 114 | 12.18 | 585 |  | 0.9 | ± | 0.6 | 0.4 | ± | 0.4 | 0.6 | ± | 0.3 | 0.4 | ± | 0.2 |  | **0.50** | **0.70** | **0.39** |  | 0.08 | 0.39 | **0.03** |
| 100 | 11.39 | 607 |  | 3.6 | ± | 1.0 | 2.8 | ± | 1.0 | 2.9 | ± | 0.8 | 2.7 | ± | 0.5 |  | 0.78 | 0.80 | **0.75** |  | 0.08 | 0.20 | **0.03** |
| 165 | 17.33 | 955 |  | 5.1 | ± | 2.8 | 3.1 | ± | 2.0 | 2.9 | ± | 1.6 | 2.1 | ± | 1.1 |  | **0.61** | **0.56** | **0.41** |  | 0.09 | 0.14 | **< 0.01** |
| 2 | 1.25 | 795 |  | 1.7 | ± | 0.5 | 1.4 | ± | 0.4 | 1.1 | ± | 0.6 | 0.8 | ± | 0.7 |  | 0.80 | **0.65** | **0.48** |  | 0.10 | **0.03** | **< 0.01** |
| 115 | 12.13 | 1172 |  | 1.5 | ± | 1.0 | 0.8 | ± | 0.7 | 1.1 | ± | 0.6 | 0.6 | ± | 0.3 |  | **0.52** | **0.72** | **0.41** |  | 0.10 | 0.46 | **0.04** |
| 1 | 1.24 | 779 |  | 5.9 | ± | 1.7 | 4.9 | ± | 1.6 | 3.6 | ± | 2.2 | 1.9 | ± | 1.5 |  | 0.83 | **0.61** | **0.33** |  | 0.18 | **0.03** | **< 0.01** |
| 10 | 1.31 | 729 |  | 2.6 | ± | 1.2 | 1.9 | ± | 0.3 | 2.1 | ± | 0.5 | 1.3 | ± | 0.6 |  | 0.75 | 0.82 | **0.53** |  | 0.18 | 0.46 | **0.02** |
| 11 | 1.32 | 387 |  | 12.5 | ± | 3.0 | 10.9 | ± | 1.3 | 11.8 | ± | 1.9 | 9.1 | ± | 2.5 |  | 0.87 | 0.94 | **0.73** |  | 0.19 | 0.66 | **0.01** |
| 21 | 5.19 | 1295 |  | 1.7 | ± | 0.6 | 1.4 | ± | 0.4 | 1.6 | ± | 0.7 | 1.2 | ± | 0.7 |  | 0.84 | 0.91 | **0.68** |  | 0.24 | 0.65 | **0.04** |
| 9 | 1.31 | 341 |  | 12.5 | ± | 3.2 | 11.1 | ± | 1.3 | 11.7 | ± | 1.8 | 9.4 | ± | 2.5 |  | 0.89 | 0.94 | **0.75** |  | 0.30 | 0.65 | **0.03** |
| 70 | 9.76 | 803 |  | 5.3 | ± | 1.9 | 5.4 | ± | 2.7 | 5.2 | ± | 0.8 | 3.3 | ± | 2.9 |  | 1.02 | 0.98 | **0.63** |  | 0.93 | 0.91 | **0.05** |

Compounds included here had both statistical significance compared to healthy trees (bold orange, α = 0.05; light orange, α < 0.01) in at least one of the decline categories and had at least 25% lower peak area (bold blue; light blue indicates < 50% healthy peak area; red font indicates > 125% healthy peak area; light red indicates > 150% healthy peak area).
