# Supplementary material for: Detection of Emerald Ash Borer Infestations in Living Green Ash by Noninvasive Electronic-Nose Analysis of Wood Volatiles
Source: Biosensors (Basel). 2019 Oct 13;9(4):123. doi: 10.3390/bios9040123 (PMC6956047; doi:10.3390/bios9040123)
Supplement: Supplementary file 1 [file biosensors-09-00123-s001.zip › biosensors-608360-supplementary/Table S3 Supplemental materials - LC-MS 3.docx]

| **Table S3.** LC-MS peak areas for green ash bark methanol-extractable contents that were significantly increased by EAB infestation. | | | | | | | | | | | | | | | | | | | | | | | |
| --- | --- | --- | --- | --- | --- | --- | --- | --- | --- | --- | --- | --- | --- | --- | --- | --- | --- | --- | --- | --- | --- | --- | --- |
|  |  |  |  | Decline class (LCMS signal intensity x 100,000) | | | | | | | | | | | |  | Fold difference | | |  | P | | |
| **No.** | **RT** | **m/z** |  | 1 | | | 2 | | | 3 | | | 4 | | |  | 2 | 3 | 4 |  | 1 v 2 | 1 v 3 | 1 v 4 |
| 63 | 9.07 | 521 |  | 2.1 | ± | 1.1 | 1.0 | ± | 0.3 | 2.3 | ± | 1.2 | 3.8 | ± | 2.7 |  | **0.50** | 1.09 | **1.82** |  | **0.02** | 0.75 | **0.02** |
| 5 | 1.3 | 181 |  | 2.5 | ± | 1.0 | 3.5 | ± | 1.4 | 4.1 | ± | 1.3 | 4.8 | ± | 1.4 |  | **1.41** | **1.65** | **1.91** |  | **0.04** | **< 0.01** | **< 0.01** |
| 134 | 13.7 | 675 |  | 4.3 | ± | 1.4 | 5.6 | ± | 1.7 | 4.2 | ± | 1.4 | 6.2 | ± | 2.4 |  | **1.31** | 0.97 | **1.44** |  | **0.04** | 0.89 | **0.01** |
| 75 | 10.09 | 837 |  | 1.6 | ± | 0.7 | 2.0 | ± | 0.4 | 1.8 | ± | 0.7 | 2.6 | ± | 1.2 |  | **1.27** | 1.14 | **1.64** |  | 0.11 | 0.55 | **< 0.01** |
| 111 | 11.89 | 595 |  | 0.1 | ± | 0.0 | 0.1 | ± | 0.0 | 0.2 | ± | 0.0 | 0.1 | ± | 0.1 |  | 0.82 | **1.39** | 1.02 |  | 0.14 | **0.02** | 0.88 |
| 79 | 10.49 | 521 |  | 0.1 | ± | 0.0 | 0.1 | ± | 0.0 | 0.2 | ± | 0.0 | 0.1 | ± | 0.1 |  | 0.76 | **1.65** | 1.16 |  | 0.19 | **< 0.01** | 0.44 |
| 38 | 6.71 | 583 |  | 4.9 | ± | 1.6 | 4.1 | ± | 1.2 | 7.6 | ± | 2.2 | 6.3 | ± | 2.1 |  | 0.83 | **1.54** | **1.28** |  | 0.22 | **< 0.01** | 0.08 |
| 160 | 17.25 | 701 |  | 0.9 | ± | 0.6 | 0.6 | ± | 0.6 | 1.6 | ± | 0.7 | 0.7 | ± | 0.6 |  | **0.68** | **1.85** | 0.82 |  | 0.31 | **0.04** | 0.56 |
| 171 | 17.47 | 715 |  | 2.2 | ± | 1.6 | 1.6 | ± | 1.3 | 4.2 | ± | 2.6 | 2.5 | ± | 2.2 |  | **0.72** | **1.86** | 1.12 |  | 0.35 | **0.05** | 0.71 |
| 130 | 13.44 | 645 |  | 1.3 | ± | 0.5 | 1.5 | ± | 0.6 | 1.1 | ± | 0.2 | 1.8 | ± | 0.6 |  | 1.17 | 0.87 | **1.42** |  | 0.37 | 0.55 | **0.03** |
| 167 | 17.38 | 911 |  | 2.1 | ± | 1.1 | 1.8 | ± | 0.8 | 3.8 | ± | 0.8 | 1.9 | ± | 1.0 |  | 0.84 | **1.81** | 0.90 |  | 0.46 | **< 0.01** | 0.65 |
| 163 | 17.34 | 735 |  | 2.2 | ± | 0.9 | 2.5 | ± | 1.6 | 3.2 | ± | 0.2 | 2.3 | ± | 1.1 |  | 1.16 | **1.46** | 1.06 |  | 0.48 | **0.04** | 0.78 |
| 14 | 4.43 | 407 |  | 3.7 | ± | 2.1 | 3.2 | ± | 1.0 | 5.8 | ± | 0.4 | 7.4 | ± | 2.8 |  | 0.85 | **1.56** | **2.00** |  | 0.51 | 0.06 | **< 0.01** |
| 159 | 17.24 | 659 |  | 0.7 | ± | 0.4 | 0.8 | ± | 0.6 | 1.3 | ± | 0.6 | 0.8 | ± | 0.5 |  | 1.17 | **1.94** | 1.18 |  | 0.53 | **< 0.01** | 0.48 |
| 162 | 17.28 | 689 |  | 4.3 | ± | 1.8 | 4.7 | ± | 2.7 | 6.5 | ± | 0.6 | 4.5 | ± | 2.0 |  | 1.10 | **1.52** | 1.06 |  | 0.63 | **0.02** | 0.75 |
| 173 | 17.56 | 729 |  | 0.9 | ± | 0.4 | 1.0 | ± | 0.7 | 2.7 | ± | 1.4 | 1.0 | ± | 0.9 |  | 1.11 | **3.00** | 1.15 |  | 0.63 | **< 0.01** | 0.59 |
| 54 | 8.37 | 581 |  | 1.8 | ± | 0.9 | 1.8 | ± | 0.9 | 4.6 | ± | 1.9 | 2.2 | ± | 1.3 |  | 0.98 | **2.48** | 1.18 |  | 0.94 | **< 0.01** | 0.46 |
| 13 | 2.6 | 479 |  | 3.0 | ± | 1.1 | 3.0 | ± | 0.6 | 4.6 | ± | 1.4 | 3.5 | ± | 1.1 |  | 0.99 | **1.53** | 1.16 |  | 0.96 | **0.02** | 0.35 |
| 16 | 4.6 | 461 |  | 9.6 | ± | 3.1 | 9.6 | ± | 1.3 | 13.2 | ± | 3.5 | 11.2 | ± | 3.5 |  | 1.00 | **1.37** | 1.16 |  | 0.99 | **0.05** | 0.27 |

Compounds included here were more abundant than in healthy trees with statistical significance (bold orange, α = 0.05; light orange, α < 0.01) in at least one of the decline categories and had at least 25% greater peak area (bold red; light red indicates > 150% healthy peak area; blue font indicates < 75% healthy peak area; light blue indicates < 50% healthy peak area).
